# Design and Implementation of an Enhanced Matched Filter for Sidelobe Reduction of Pulsed Linear Frequency Modulation Radar

**DOI:** 10.3390/s21113835

**Published:** 2021-06-01

**Authors:** Ahmed Azouz, Ashraf Abosekeen, Sameh Nassar, Mohamed Hanafy

**Affiliations:** 1 Electrical Engineering Branch, Military Technical College, Kobry El-Kobba, Cairo 11766, Egypt; a.abosekeen@ieee.org (A.A.); mehanafy@mtu.edu (M.H.); 2 Mobile Multi-Sensor Systems Research Group, University of Calgary, Calgary, AB T2N 1N4, Canada; snassar@ucalgary.ca

**Keywords:** sidelobe reduction filter, linear frequency modulated, enhanced matched filter

## Abstract

Pulse compression techniques are commonly used in linear frequency modulated (LFM) waveforms to improve the signal-to-noise ratios (SNRs) and range resolutions of pulsed radars, whose detection capabilities are affected by the sidelobes. In this study, a sidelobe reduction filter (SRF) was designed and implemented using software defined radio (SDR). An enhanced matched filter (EMF) that combines a matched filter (MF) and an SRF is proposed and was implemented. In contrast to the current commonly used approaches, the mathematical model of the SRF frequency response is extracted without depending on any iteration methods or adaptive techniques, which results in increased efficiency and computational speed for the developed model. The performance of the proposed EMF was verified through the measurement of four metrics, including the peak sidelobe ratio (PSLR), the impulse response width (IRW), the mainlobe loss ratio (MLR), and the receiver operational characteristics (ROCs) at different SNRs. The ambiguity function was then used to characterize the Doppler effect on the designed EMF. In addition, the detection of single and multiple targets using the proposed EMF was performed, and the results showed that it overcame the masking problem due to its effective reduction of the sidelobes. Hence, the practical application of the EMF matches the performance analysis. Moreover, when implementing the EMF proposed in this paper, it outperformed the common MF, especially when detecting targets moving at low speeds and having small radar cross-sections (RCS), even under severe masking conditions.

## 1. Introduction

**Figure 1:**
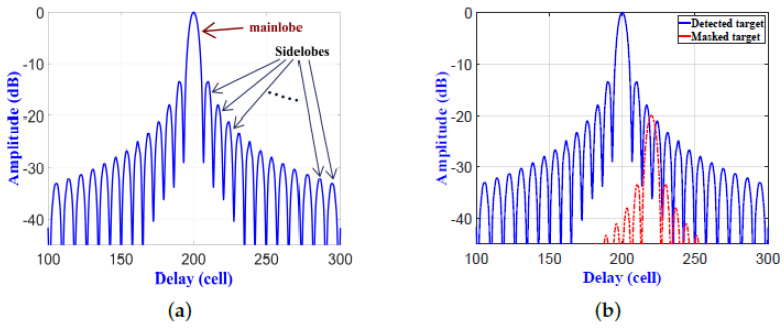
(**a**) Mainlobe and sidelobes of Matched Filter (MF) for a stationary target. (**b**) Masked target within the sidelobes of a detected target.

Pulse compression techniques for linear frequency modulation (LFM) waveforms are commonly used with surveillance and tracking radars. Since the LFM waveforms have high Doppler tolerance, LFM pulse compression is accomplished by applying frequency modulation to a long pulse before transmission. A pulse compression technique is considered an essential feature in radar systems [1,2], where it is used for wide pulses with low peak power to achieve a detection range and resolution that are provided by narrow pulses with high peak power [3]. To achieve high range resolution, the compressed wide pulse should have large spectral bandwidth. That same concept used for radar LFM waveforms has been applied to ultrasonic guided waves [4], active thermal non-destructive testing [5], and truncated-correlation photothermal coherence tomography [6]. A matched filter (MF) is usually used to compress the received LFM signal and to improve the signal-to-noise ratio (SNR) by adding compression gain to the received pulse [7]. The output signal from the MF contains a peak spike, called the mainlobe, along with surrounding low spikes, called sidelobes, as shown in Figure 1a [8]. Detection of a single target in the line-of-sight of the radar using MF should be a simple process due to the absence of clutter. However, the signal received from a single target with a low radar cross-section (RCS) can be dissimulated either by other signals due to various sources of clutter, including clouds, mountains, and large buildings; or by a neighboring target with a large RCS, as shown in Figure 1b [9]. This is due to the fact that the peak sidelobe ratio (PSLR) is close to the nominal value of −13.2 dB for an LFM waveform [9]; i.e., the amplitude of the first sidelobe is $(10^{-13.2/20})\times 100\approx 21.88$% of the mainlobe. This was the main idea that motivated us to design and implement an effective sidelobe reduction filter for pulsed LFM radar that eliminates the sidelobes and solve the target’s dissimulating problem. Typical scenarios include detecting vehicles beside a mountain or hill, and detecting unmanned aerial vehicles (UAVs) masked by airplanes having large RCSs.

Several methods are used for sidelobe reduction: In [10], the complementary method was applied for LFM waveform to completely remove the sidelobes, where sequential complementary codes (pulse-to-pulse) are transmitted sequentially. However, this method has many barriers in real applications [11,12]. Other methods use a waveform that has good autocorrelation function (ACF) properties with low autocorrelation sidelobe levels and reduced impulse response width (IRW). Such a waveform can be obtained by using binary codes [13], polyphase codes [14], Costas codes [15], and nonlinear frequency modulation (FM) [16,17,18]. Another method uses powerful convex optimization to generate a waveform that has a strong ACF and a PSLR of around −46 dB; see [19]. A new polyphase-code with a good ACF with a PSLR value of around −40.2 dB was introduced in [20]. Although these methods generate strong ACFs, they are limited for sidelobe reduction to specific waveforms generated with optimized parameters only. Moreover, the sidelobe reduction efficiency is affected by any change in the parameters of the waveform, which is considered as a main drawback of these methods. Another technique that also has a good ACF is applied to sonar waves, aiming to reduce levels of the sidelobes by using LFM–Costas and generalized sinusoidal frequency-modulated trains which depend on the genetic algorithm [21]. Depth and resolution optimization for thermal wave radar imaging by generating good ACFs using frequency-phase modulated waveforms was performed in one study [22], and adjustable frequency bandwidths and frequency chirp repetition rates were optimized for LFM waveforms in [23].

An alternate solution to the sidelobe reduction by multiplying the output signals from the MF with a prober window function, such as a Blackman, Flattop, Hanning, or Hamming one, is discussed in [24]. In general, the Hamming window (HW) usually has the best performance among the aforementioned techniques, since it reduces the level of the sidelobes to lower than −40 dB. However, the IRW will increase, leading to a degraded resolution; e.g., IRW=1.33 bin [25]. Additionally, the level of the mainlobe will be reduced. Window function optimization for sidelobe reduction after chirp signal compression and for the design of FM signals was covered in [26]. In an ultrasonic nondestructive evaluation using LFM, combined windowed optimization tended to be the best solution for narrowband systems when very low sidelobe levels were needed, at the cost of reduced IRW [27]. The frequency domain weighting function for sidelobe reduction to LFM by using double spatially variant apodization was applied in [28]. The main drawback of the aforementioned windowing functions is the reduction of both the resolution and mainlobe energy [9,29].

Other research approaches to reduce the sidelobe level include: the mismatched filter (MMF), the Rihaczek and Golden (R-G) filter, and adaptive algorithms. In the first approach, the MMF is designed by using the least-mean-square (LMS) criterion, where the MMF follows the MF with a multiple-stage finite impulse response (FIR) or infinite impulse response (IIR) filter to reduce the sidelobe level [30]. In [31], MMF minimizes the integrated sidelobe level (ISL) by minimizing the sidelobes’ energy. In [32], MMF was applied for LFM waveforms, resulting in a reduction of both mainlobe energy and resolution. In [33], a proposed least-square MMF (LS-MMF) was applied to an FM waveform, resulting in a PSLR enhancement of 8 dB. In [34], convex optimization was used with MMF for a polyphase Barker with a PSLR of −46 dB. On the other hand, the second approach applied an R-G filter [35], which operated in the frequency domain, to reduce the complexity of a digital processor. An R-G filter improved by reducing its complexity based on a linear programming algorithm is introduced in [36]. A modified R-G filter for binary codes that reduces the sidelobe level to −40 dB through an optimization operation is discussed in [37]. However, the R-G filter has not been implemented for the LFM waveform.

In the third approach, several adaptive algorithms are used to reduce the sidelobes in range direction. These adaptive algorithms include: the Wiener filter, the CLEAN algorithm, and the adaptive pulse compression (APC) algorithm. The Wiener filter relies on space-time adaptive processing to maximize the signal-to-interference ratio through clutter or jamming interference cancellation [38]. The Wiener filter is also used for continuous LFM waveforms in range direction [39,40]. The CLEAN algorithm removes interference from large scatterer by adaptively removing its sidelobes via sequential subtraction process [41]. The CLEAN algorithm was applied in [42] to remove the sidelobe interferences when expressing target range profiles for wideband binary coding signals. The CLEAN algorithm has one main drawback, which is that the Doppler effect must be removed before using the MF, which is a complex process. In the APC algorithm, the sidelobes in the range direction are reduced by applying a unique pulse compression filter for each range cell [43]. An APC technique depending on the reiterative minimum mean-square error (RMMSE) algorithm was used in [44]. The practical execution of this algorithm is limited by highly complex calculations and sensitivity to Doppler mismatching. A modified MMF filter combined with APC and least-squares for polyphase-code FM is presented in [45]. Recently, an iterative deconvolution time-reversal method that can remove the blur caused by the channel using the time-reversal method was shown in [46].

In the inverse filter technique for the LFM waveform, implemented in [47], the sidelobe reduction is performed for the case of a zero-centered frequency LFM, where only the peak value of the mainlobe will pass. This technique was then applied in [48] for synthetic aperture radar (SAR) processing in range direction. Additionally, sidelobe reduction Barker–LFM was utilized in [49]. The sibelobe reduction for squarer length polyphase codes by using inverse filter technique was employed in [50,51]. For any type of length of waveform, general sidelobe reduction was executed in [52] depending on phases of the waveform.

In this paper, all the calculations associated with the sidelobe reduction filter of the designed and implementated enhanced matched filter (EMF) are dependent on the parameters of the waveform, and hence the time of calculation is considerably reduced compared to the phase-dependent technique used in [52]. Additionally, compared to the results obtained in [47], which is applied only for the zero-centered frequency LFM, the SNR is enhanced due to the reduced bandwidth of the sidelobe reduction filter of the EMF. Additionally, the general formula that represents the filter is improved for the cases of zero-centered and zero-started frequency LFM.

Here, a sidelobe reduction filter (SRF) is modeled and implemented using software defined radio (SDR). The proposed EMF combines the MF and the SRF. The performance analysis of the filter was verified by the measurement of four different metrics including the PSLR, IRW, mainlobe loss ratio (MLR), and receiver operational characteristics (ROCs) at different SNRs. The designed EMF was then tested for the detection of single and multiple targets using indoor and outdoor datasets. The obtained results showed that the proposed EMF successfully overcame the masking problem due to its effective reduction of sidelobes.

Therefore, the main contributions in this paper include:

1. An EMF, combining the MF and the SRF, for LFM waveforms is proposed.
2. A derived formula for the frequency response of the SRF is presented, depending on the parameters of the LFM signal and without using any iteration methods or adaptive techniques as used previously in the literature, where the operational principle of the MMF, R-G, Wiener, CLEAN, and APC algorithms depends on either an iteration using least-squares, or optimization of an algorithm parameter to reduce the sidelobes. Additionally, other techniques combine both iteration and optimization methods to reduce the sidelobes [45].
3. Since the windowing functions and MMF [8] increase the IRW more than an MF, the proposed EMF keeps the IRW equivalent to that resulting from an MF, hence reserving the resolution. Additionally, it provides a considerable reduction in sidelobes.
4. The proposed EMF maintains the peak level of the mainlobe as MF does, while the windowing function, MMF [8], and APC [53], reduce the peak value.

The paper is organized as follows: Section 2 presents the structure of the proposed EMF and the mathematical model of the SRF. Section 3 investigates the performance of the developed EMF, and also comparing its performance with previous work in the field. Section 4 illustrates the real implementation and testing of the LFM radar with the proposed EMF. Finally, conclusions are presented in Section 5.

## 2. Derivation of the Sidelobe Reduction Filter (SRF)

**Figure 2:**
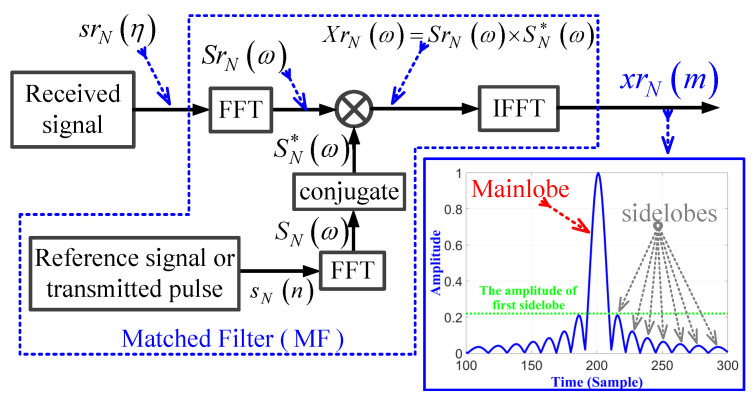
Block diagram of the conventional MF.

The usage of the conventional MF in processing the received discrete LFM signal in the frequency domain is shown in Figure 2. The general form of the transmitted single-pulsed LFM waveforms in discrete time form $s_{N}\left(n\right)$, is expressed by [54]:

(1) $$s_{N}\left(n\right)=A\exp\left(j\omega_{o}n+j\;k\;\pi \;n^{2}\right)\;\;\;\;\;n=\left\{\begin{array}{c} -N/2⩽n⩽N/2\;\;\;\;\;\;\mathrm{zero-centered}\;\mathrm{frequency}\\ \;\;\;\;\;0\;\;⩽n⩽N-1\;\;\;\;\;\mathrm{zero-started}\;\mathrm{frequency} \end{array}\right.\;$$

where $\omega_{o}=2\pi f_{o}/f_{s}$ is the discrete angular frequency shift, with $f_{s}$ and $f_{o}$ being sampling frequency and frequency shift, respectively; *A* is the amplitude; $k=B_{r}/\left(Nf_{s}\right)$ is the LFM chirp rate, where $B_{r}$ is the chirp bandwidth, and *N* is the number of samples in the transmitted LFM waveform pulse.

The received signal $sr_{N}\left(\eta \right)$ that is acquired from a single target can be presented as:

(2) $$sr_{N}\left(\eta \right)=A_{r}\;s_{N}\left(\eta -n_{d}\right),$$

where $A_{r}$ is the amplitude of the received signal, $n_{d}$ is the delay received signal from target due to two-way traveling, and $\eta =\{0,1,2,\cdots ,N_{r}-1\}$ is the received samples, with $N_{r}$ total samples in range direction. In the frequency domain, both the transmitted signal $s_{N}\left(n\right)$ and the received signal $sr_{N}\left(\eta \right)$ are converted using the fast Fourier transform (FFT) to be $S_{N}\left(\omega \right)$ and $Sr_{N}\left(\omega \right)$, respectively. $Sr_{N}\left(\omega \right)$ and its conjugate, $S_{N}^{*}\left(\omega \right)$, are multiplied together to obtain the correlation signal in frequency domain $Xr_{N}\left(\omega \right)$. The resulting signal $Xr_{N}\left(\omega \right)$ is converted into time domain signal $xr_{N}\left(m\right)$ by using the inverse FFT (IFFT), which is the output of the MF, as shown in Figure 2.

The output $xr_{N}\left(m\right)$ of the MF is a compressed signal with m=0,1,⋯,M−1, where $M=N+N_{r}-1$ is the total number of samples at MF output. The amplitude response of MF for pulsed LFM is plotted for $f_{s}=200$ MHz, $B_{r}=20$ MHz, N=500, $N_{r}=1024$, and $n_{d}=200$. The sidelobes at the MF output, due to the compression process, have a significant effect on the detection of low RCS targets. The first sidelobe peak value represents 21% of the mainlobe peak value.

**Figure 3:**
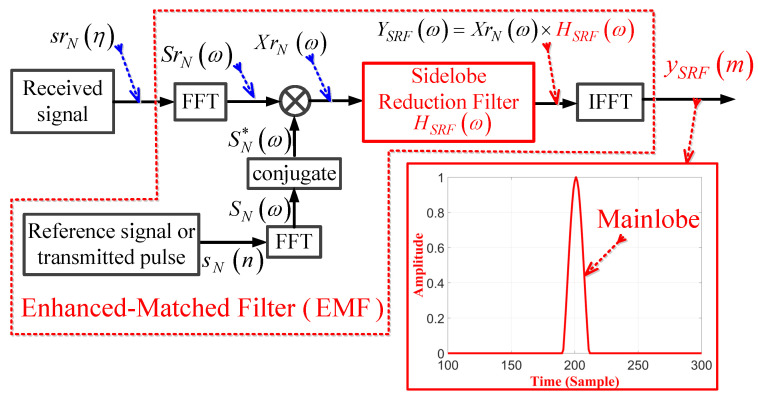
Block diagram of the proposed EMF.

To reduce these sidelobes, we propose an EMF which is shown in Figure 3. The construction of the EMF includes a basic MF in addition to the SRF and IFFT blocks. Here $Xr_{N}\left(\omega \right)$ is multiplied by $H_{SRF}\left(\omega \right)$, which is the frequency response of the SRF, to obtain $Y_{SRF}\left(\omega \right)$. IFFT is then applied to $Y_{SRF}\left(\omega \right)$ to obtain $y_{SRF}\left(m\right)$.

**Figure 4:**
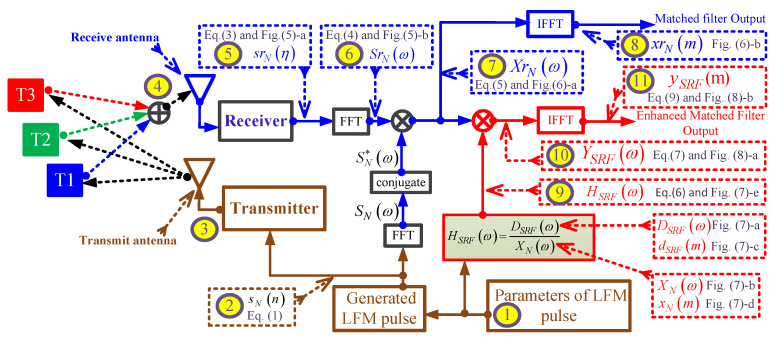
A detailed flowchart of the proposed EMF dealing with three targets.

**Figure 5:**
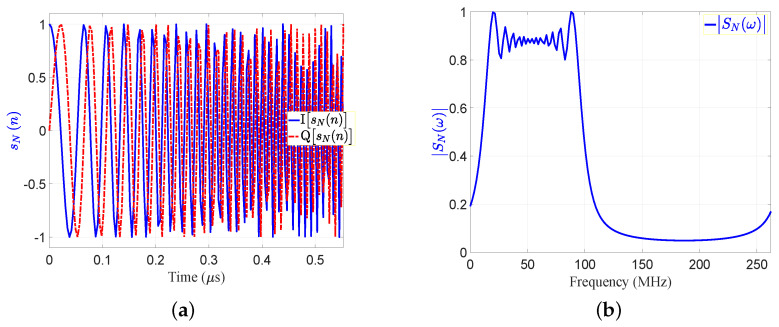
The transmitted signal in time and frequency domains:(**a**) $s_{N}\left(n\right)$; (**b**) $\left|S_{N}\left(\omega \right)\right|$.

**Figure 6:**
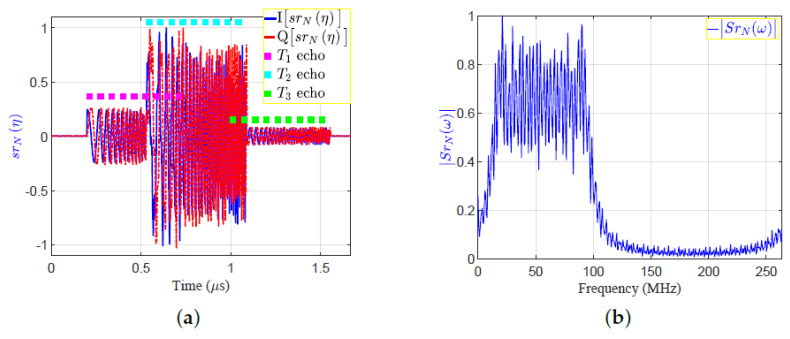
The received signal in time and frequency domains: (**a**) $sr_{N}\left(\eta \right)$; (**b**) $\left|Sr_{N}\left(\omega \right)\right|$.

**Figure 7:**
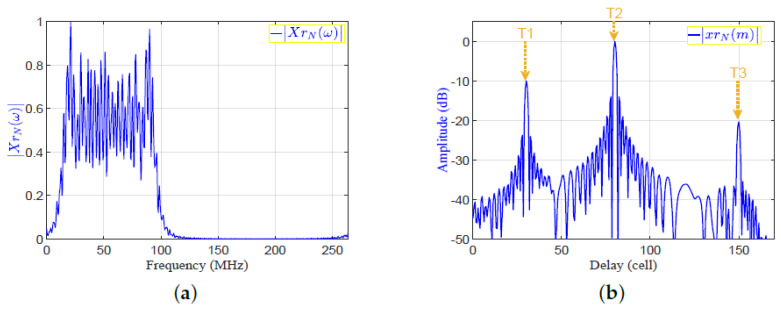
The matched filter output in frequency and time domains: (**a**)$\left|Xr_{N}\left(\omega \right)\right|$; (**b**)$\left|xr_{N}\left(m\right)\right|$.

**Figure 8:**
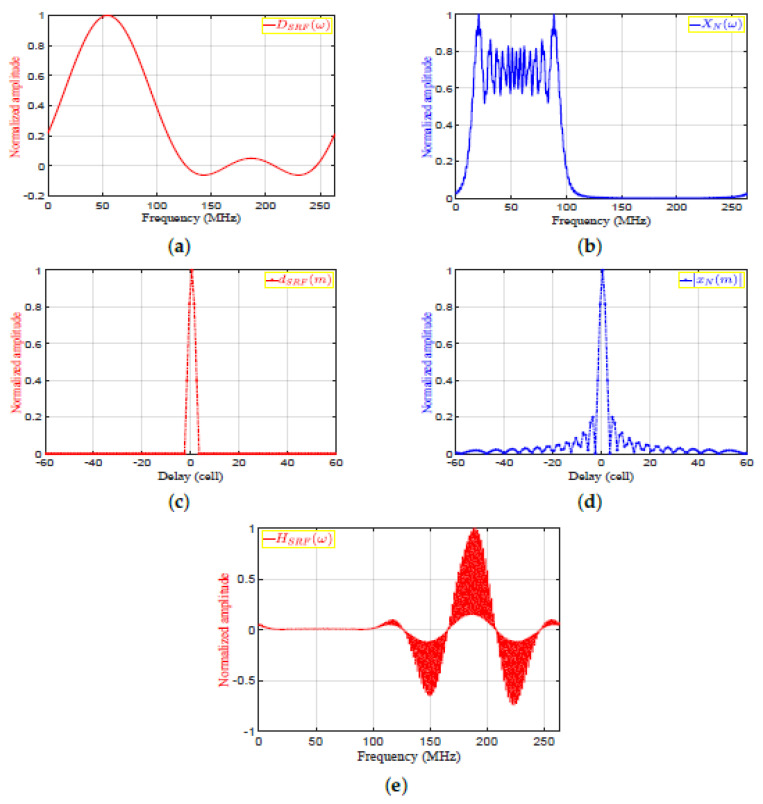
The sidelobe reduction filter: (**a**) $D_{SRF}\left(\omega \right)$, (**b**) $X_{N}\left(\omega \right)$, (**c**) $d_{SRF}\left(m\right)$, (**d**) $\left|x_{N}\left(m\right)\right|$, (**e**) $H_{SRF}\left(\omega \right)$.

**Figure 9:**
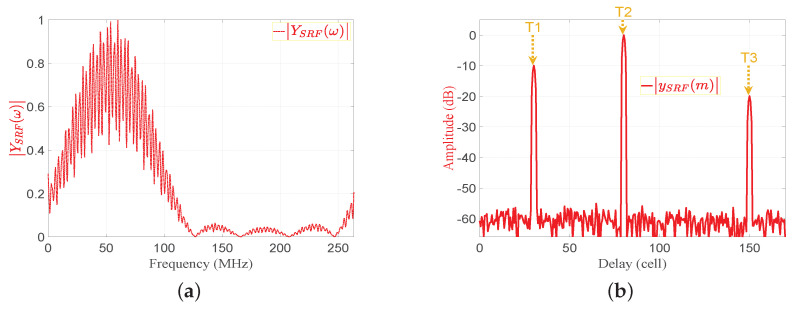
The enhanced matched filter output in frequency and time domains: (**a**) $Y_{SRF}\left(\omega \right)$, (**b**) $xr_{N}\left(m\right)$, and $y_{SRF}\left(m\right)$.

Figure 4 clarifies the concept of the SRF using the proposed EMF according to the following scenario:

1. The parameters for pulsed LFM include: $f_{s}=264$ MHz, $B_{r}=90$ MHz, $f_{o}=10$ Mhz, N=147 sample size, and $N_{r}=441$ sample size.
2. These parameters are used in Equation (1) to generate the LFM waveform.
3. The generated LFM waveform is then up-converted by the transmitter, and is then propagated by means of the transmit antenna towards three targets (T1:T3). The time domain of base-band transmitted signal, $s_{N}\left(n\right)$, and the absolute of its FFT, $S_{N}\left(\omega \right)$, in Figure 5a,b, respectively. The transmitted signal, $s_{N}\left(n\right)$, is a complex signal that contains the In-phase (I) and quadrature phase (Q) components.
4. The three reflected echoes from the three targets are summed up at the receive antenna; then the receiver down-converts the received signal into a baseband. The amplitude of the reflected echoes, having different RCSs and being located at different ranges, have normalized amplitudes: $A_{r_{1}}=0.3162$, $A_{r_{2}}=1$, and $A_{r_{3}}=0.1$. Consequently, the received signals are time delayed by numbers of samples: $n_{d_{1}}=53$, $n_{d_{2}}=141$, and $n_{d_{3}}=265$, which correspond to the ranges: $d_{1}=30$ m, $d_{2}=80$ m, and $d_{3}=150$ m, respectively.
5. The received signal after down-conversion at receiver, $sr_{N}\left(\eta \right)$ is the sum of echo signal from the three targets, and is expressed by:
  (3) $$sr_{N}\left(\eta \right)=A_{r_{1}}\;s_{N}\left(\eta -n_{d_{1}}\right)+A_{r_{2}}\;s_{N}\left(\eta -n_{d_{2}}\right)+A_{r_{3}}\;s_{N}\left(\eta -n_{d3}\right)$$
6. In the frequency domain, the received signal $Sr_{N}\left(\omega \right)$ is expressed by:
  (4) $$\begin{array}{cc} Sr_{N}\left(\omega \right)= & A_{r_{1}}\;S_{N}\left(\omega \right)\;\exp\left(-j\omega n_{d_{1}}\right)+A_{r_{2}}\;S_{N}\left(\omega \right)\;\exp\left(-j\omega n_{d_{2}}\right)\\ \; & \;\;\;\;\;\;\;\;\;\;\;\;\;\;\;\;\;\;\;\;\;\;\;\;\;\;\;\;\;\;\;+A_{r_{3}}\;S_{N}\left(\omega \right)\;\exp\left(-j\omega n_{d3}\right) \end{array}$$
  The time domain of baseband received complex signal, $sr_{N}\left(\eta \right)$, and the absolute of its FFT, $\left|Sr_{N}\left(\omega \right)\right|$, are shown in Figure 6a,b, respectively.
7. The conjugate of the transmitted signal $S_{N}^{*}\left(\omega \right)$ is then multiplied by the received signal, $Sr_{N}\left(\omega \right)$, to get the correlation signal in frequency domain, $Xr_{N}\left(\omega \right)$:
  (5) $$\begin{array}{cc} Xr_{N}\left(\omega \right) & =[A_{r_{1}}\;S_{N}\left(\omega \right)\;\exp\left(-j\omega \;n_{d_{1}}\right)+A_{r_{2}}\;S_{N}\left(\omega \right)\;\exp\left(-j\omega \;n_{d_{2}}\right)\\ \; & \;\;\;\;\;\;\;\;\;\;\;\;\;\;\;\;\;\;\;\;\;\;\;\;\;\;\;\;\;\;\;\;\;\;\;\;+A_{r_{3}}\;S_{N}\left(\omega \right)\;\exp\left(-j\omega \;n_{d3}\right)]\times S_{N}^{*}\left(\omega \right)\\ \; & =A_{r_{1}}\;X_{N}\left(\omega \right)\;\exp\left(-j\omega \;n_{d_{1}}\right)+A_{r_{2}}\;X_{N}\left(\omega \right)\;\exp\left(-j\omega \;n_{d_{2}}\right)\\ \; & \;\;\;\;\;\;\;\;\;\;\;\;\;\;\;\;\;\;\;\;\;\;\;\;\;\;\;\;\;\;\;\;\;\;\;\;+A_{r_{3}}\;X_{N}\left(\omega \right)\;\exp\left(-j\omega \;n_{d3}\right) \end{array}$$
  where $X_{N}\left(\omega \right)=S_{N}\left(\omega \right)\times S_{N}^{*}\left(\omega \right)$ is the Fourier transform of the autocorrelation function of the transmitted signal.
8. Applying IFFT to $X_{N}\left(\omega \right)$ to get $x_{N}\left(m\right)$, which is the output of the MF in time domain. The absolutes of $Xr_{N}\left(\omega \right)$ and $xr_{N}\left(m\right)$ are shown in Figure 7a,b, respectively.
9. The proposed frequency response of the SRF, $H_{SRF}\left(\omega \right)$, is given by:
  (6) $$H_{SRF}\left(\omega \right)=\frac{D_{SRF}\left(\omega \right)}{X_{N}\left(\omega \right)}$$
  where $D_{SRF}\left(\omega \right)$ is the frequency response of the desired output. $D_{SRF}\left(\omega \right)$ is obtained by selecting the mainlobe of $x_{N}\left(m\right)$ and zeroing the rest of samples to obtain $d_{SRF}\left(m\right)$, and then applying FFT to $d_{SRF}\left(m\right)$. The desired outputs in frequency and time domains, $D_{SRF}\left(\omega \right)$ and $d_{SRF}\left(m\right)$, are shown in Figure 8a,c, respectively. The autocorrelation functions of the transmitted signal in frequency and time domains, $X_{N}\left(\omega \right)$ and $x_{N}\left(m\right)$, are shown in Figure 8b,c, respectively. Figure 8e, is shown the frequency response of the SRF, $H_{SRF}\left(\omega \right)$. The denominator, $X_{N}\left(\omega \right)$, represents the MF output in frequency domain for a single target. $X_{N}\left(\omega \right)$, $D_{SRF}\left(\omega \right)$, and $H_{SRF}\left(\omega \right)$ are real signals as shown in Figure 8a,b and e, respectively. Detailed derivations of the general form of the $H_{SRF}\left(\omega \right)$ are shown in Appendix A for even and odd numbers of samples. Accordingly, the result of applying on the MF output is the reduction of the sidelobes without considering the transmitted or received LMF waveform.
10. The output of the SRF, $Y_{SRF}\left(\omega \right)$, is given by:
  (7) $$\begin{array}{cc} Y_{SRF}\left(\omega \right) & =[A_{r_{1}}\;X_{N}\left(\omega \right)\;\exp\left(-j\omega n_{d_{1}}\right)+A_{r_{2}}\;X_{N}\left(\omega \right)\;\exp\left(-j\omega n_{d_{2}}\right)\\ \; & \;\;\;\;\;\;\;\;\;\;\;\;\;\;\;\;\;\;\;\;\;\;\;\;\;\;\;\;\;\;\;\;\;\;+A_{r_{3}}\;X_{N}\left(\omega \right)\;\exp\left(-j\omega n_{d3}\right)]\times H_{SRF}\left(\omega \right) \end{array}$$
  From Equations (6) and (7):
  (8) $$\begin{array}{cc} Y_{SRF}\left(\omega \right) & =A_{r_{1}}\;D_{SRF}\left(\omega \right)\;\exp\left(-j\omega n_{d_{1}}\right)+A_{r_{2}}\;D_{SRF}\left(\omega \right)\;\exp\left(-j\omega n_{d_{2}}\right)\\ \; & \;\;\;\;\;\;\;\;\;\;\;\;\;\;\;\;\;\;\;\;\;\;\;\;\;\;\;\;\;\;\;\;\;\;\;\;\;+A_{r_{3}}\;D_{SRF}\left(\omega \right)\;\exp\left(-j\omega n_{d3}\right) \end{array}$$
11. Finally the output of the EMF in the time domain, $y_{SRF}\left(m\right)$, is obtained via IFFT of $Y_{SRF}\left(\omega \right)$:
  (9) $$y_{SRF}\left(m\right)=A_{r_{1}}\;d_{SRF}\left(m-n_{d_{1}}\right)+A_{r_{2}}\;d_{SRF}\left(m-n_{d_{2}}\right)+A_{r_{3}}\;d_{SRF}\left(m-n_{d3}\right)$$
  The absolutes of $Y_{SRF}\left(\omega \right)$ and $y_{SRF}\left(m\right)$ are shown in Figure 9a,b, respectively. Figure 9b shows that the PSLR corresponding to the proposed EMF is less than that of the MF output shown in Figure 7b.

The derivation of the mathematical formula for $H_{SRF}\left(\omega \right)$ is explained in detail in Appendix A. The general formulas of $H_{SRF}\left(\omega \right)$ for odd *N* and $H_{SRF,O}^{}\left(\omega \right)$, and even *N* and $H_{SRF,E}^{}\left(\omega \right)$, are given by:

(10) $$H_{SRF,O}^{}\left(\omega \right)=\frac{H_{no}\left(\omega \right)}{H_{do}\left(\omega \right)}$$

where

$$\begin{array}{cc} \; & H_{no}\left(\omega \right)=N+\sum_{a=1}^{\frac{N_{u}}{2}+1}{FO}_{N}+\sum_{a=1}^{\frac{N_{u}}{2}}{FE}_{N}\;\;,\;\;\;\;\;\;\;\;\;\;\;\;\;\;\;\;\;\;\;\;\;\;\;\;\;\;\;\;\;\;\;\;\;\;\;\;\;\;\;\;\;\;\;\;\;\;\;\;\;\;\;\;\;\;\;\;\;\;\;\;\;\;\;\;\;\;\;\;\;\;\;\;\;\;\;\;\;\;\;\;\;\;\;\\ \; & H_{do}\left(\omega \right)=N+2\cos\left(\left(N-1\right)\;\Omega_{o}\;\right)+\sum_{a=1}^{\frac{N-1}{2}}{FO}_{N}+\sum_{a=1}^{\frac{N-1}{2}-1}{FE}_{N}\;\;,\\ \; & {FO}_{N}=4\left(\sum_{b=1}^{\frac{N+1}{2}-a}O_{N}\right)\cos\left(\left(2a-1\right)\Omega_{o}\right)\;,\;\;{FE}_{N}=2\left(\sum_{b=1}^{\frac{N-1}{2}-a}E_{N}+1\right)\cos\left(2a\;\Omega_{o}\right)\;\;,\\ \; & N_{u}=\left\lfloor f_{s}/B_{r}\right\rfloor \;,\;\;O_{N}=\cos\left(\left(2a-1\right)\left(2b-1\right)\pi \;k\right)\;,\;\;E_{N}=2\cos\left(4\;\pi \;a\;b\;k\right)\;,\\ \; & \Omega_{o}=\left\{\begin{array}{c} \;\;\;\;\;\;\;\;\;\;\omega -\omega_{o}\;\;\;\;\;\;\;\;\;\;\;\;\;\;\;\;\;\;\;\;\;\;\;\;\;\;\;\mathrm{for}\;\mathrm{zero-centered}\;\mathrm{frequency}\;\mathrm{LFM}\;\mathrm{waveform}\\ \left(N-1\right)\pi k-\omega +\omega_{o}\;\;\;\;\mathrm{for}\;\mathrm{zero-started}\;\mathrm{frequency}\;\mathrm{LFM}\;\mathrm{waveform} \end{array}\right.,\\ \; & \omega =\frac{2\pi }{N_{r}}\;\;\;\left[0,1,2,......,N_{r}-2,N_{r}-1\right]\;,\;\;\mathrm{and}\;\;N_{r}\;\mathrm{is}\;\mathrm{the}\;\mathrm{total}\;\mathrm{number}\;\mathrm{of}\;\mathrm{received}\;\mathrm{samples}. \end{array}$$

(11) $$H_{SRF,E}^{}\left(\omega \right)=\frac{H_{ne}\left(\omega \right)}{\;H_{de}\left(\omega \right)}$$

where

$$\begin{array}{cc} \; & H_{ne}\left(\omega \right)=N+\sum_{a=1}^{\frac{N_{u}}{2}}{FG}_{N}+\sum_{a=1}^{\frac{N_{u}}{2}}{FP}_{N}\;\;,\;\;\;\;\;\;\;\;\;\;\;\;\;\;\;\;\;\;\;\;\;\;\;\;\;\;\;\;\;\;\;\;\;\;\;\;\;\;\;\;\;\;\;\;\;\;\;\;\;\;\;\;\;\;\;\;\;\;\;\;\;\;\;\;\;\;\;\;\;\;\;\;\;\;\;\;\;\;\;\;\;\;\;\;\;\;\;\;\;\;\;\;\;\;\\ \; & H_{de}\left(\omega \right)=N+2\cos\left(\left(N-1\right)\;\Omega_{e}\right)+\sum_{a=1}^{\frac{N}{2}-1}{FG}_{N}+\sum_{a=1}^{\frac{N}{2}-1}{FP}_{N}\;\;,\\ \; & {FG}_{N}=\left(4\sum_{b=1}^{\frac{N}{2}-a}G_{N}+2\right)\cos\left(\left(2a-1\right)\Omega_{e}\right)\;\;,\;{FP}_{N}=4\left(\sum_{b=1}^{\frac{N}{2}-a}P_{N}\right)\cos\left(2a\Omega_{e}\right)\;\;,\\ \; & G_{N}=\cos\left(\left(2a-1\right)\left(2\pi bk\right)\right)\;,\;\;P_{N}=\cos\left(2a\left(2b-1\right)\pi k\right)\;\;,\;\mathrm{and}\;\;\\ \; & \Omega_{e}=\left\{\begin{array}{c} \;\;\;\;k\pi +\omega -\omega_{o}\;\;\;\;\;\;\;\;\;\;\;\;\;\;\;\;\;\;\mathrm{for}\;\mathrm{zero-centered}\;\mathrm{frequency}\;\mathrm{LFM}\;\mathrm{waveform}\\ \left(N-1\right)\pi \;k-\omega +\omega_{o}\;\;\;\;\;\mathrm{for}\;\mathrm{zero-started}\;\mathrm{frequency}\;\mathrm{LFM}\;\mathrm{waveform} \end{array}\right. \end{array}$$

**Figure 10:**
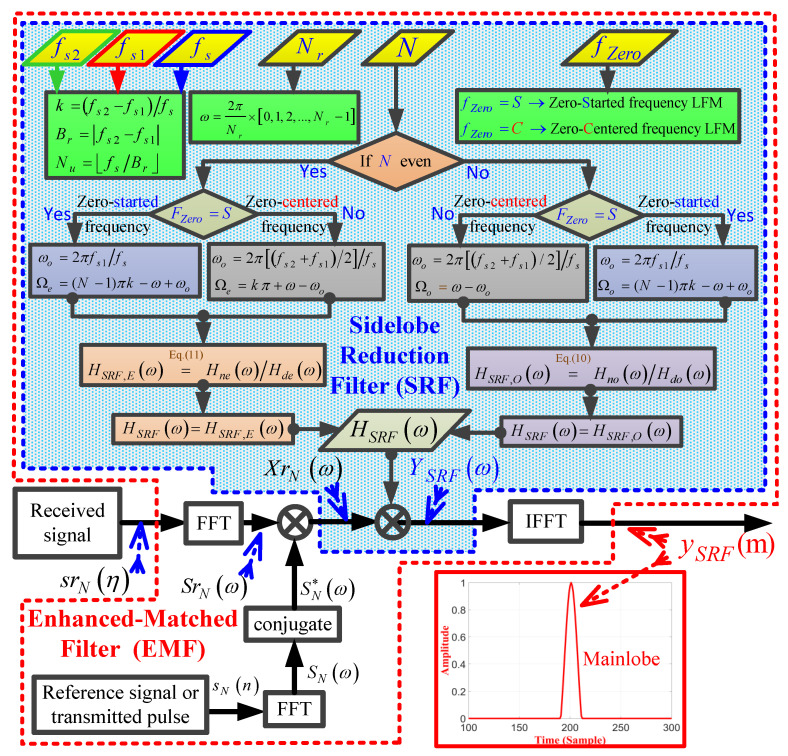
A detailed flowchart of the proposed EMF for an LFM waveform.

Figure 10 demonstrates a detailed flowchart of the proposed EMF for an LFM waveform. The main input parameters of the EMF include: the start frequency of the LFM waveform, $f_{s1}$; the stop frequency of the LFM waveform, $f_{s2}$, $f_{s}$, $N_{r}$, *N*, or $f_{Zero}$ whose value depends on whether the LFM waveform is zero-centered frequency, $f_{Zero}\;=\;C$, or zero-started frequency—$f_{Zero}\;=\;S$, $k=\left(f_{s2}-f_{s1}\right)/f_{s}$, $B_{r}=\left|f_{s2}-f_{s1}\right|$, and $\omega =\left(2\pi /N_{r}\right)\left(0,1,2,\cdots ,N_{r}-1\right)$. For $f_{Zero}\;=\;S$, $\omega_{o}=2\pi f_{s1}/f_{s}$, and for $f_{Zero}\;=\;C$, $\omega_{o}=2\pi \left[\left(f_{s2}+f_{s1}\right)/2\right]/f_{s}$. For odd *N*, the frequency response of the EMF is calculated based on Equation (10), whereas for an even *N*, the frequency response of EMF is calculated based on Equation (11).

Hence, EMF utilizes the predefined values for the input parameters of the pulsed LFM waveform without depending on any iteration methods or adaptive techniques as used in current available methods discussed earlier in Section 1 (MMF, R-G filter, Wiener filter, CLEAN filter, and APC).

## 3. Performance Analysis of the Proposed EMF

In this section, the performance of the proposed EMF is compared to the performances of two common sidelobe reduction filters: the first one is a basic MF, and the second is a Hamming filter (HF), which is a basic MF multiplied by a HW. This analysis considers a single target, and the results were verified through measurements of four different metrics, including the PSLR, IRW, MLR, and ROC at different values of SNR. The ambiguity function is then used to characterize the Doppler effect on the three filters. Then, to confirm the efficiency of the proposed EMF in solving the masking problem, its performance is compared with previous sidelobe reduction techniques, including modified MMF using APC and least-squares for polyphase-code FM, which were presented in [45].

### 3.1. Performance Analysis Considering a Single Target

**Figure 11:**
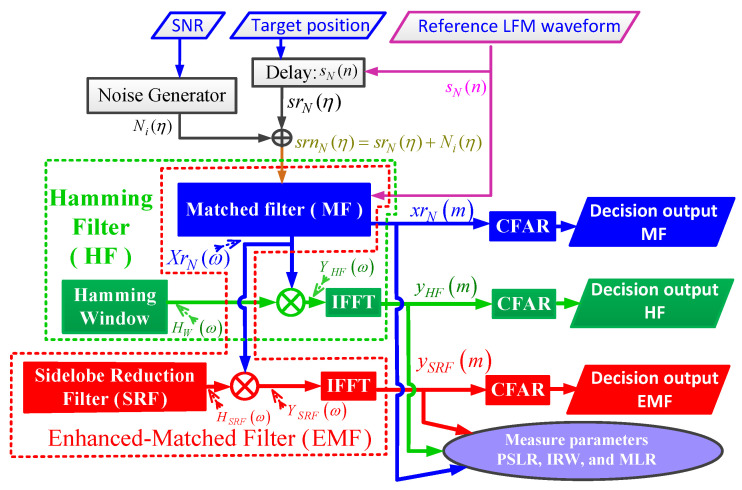
Flowchart of the performance analysis of MF, HF, and the proposed EMF for a single target.

A flowchart of the performance analysis of MF, HF, and the proposed EMF, for a single target, is shown in Figure 11.

The performance analysis was carried out through the following steps:

1. Generating the reference LFM waveform, $s_{N}\left(n\right)$, using Equation (1) with the following parameter values: target range = 200 m, $\omega_{o}=0$ rad/s, $B_{r}=90$ MHz, and $f_{s}=120$ MHz. Considering three different values for *N*, 43, 85, and 171, which correspond to three values of time bandwidth product (TBP) of the baseband LFM signal: 32, 64, and 128, respectively, where $TBP=B_{r}\times N/f_{s}$.
2. The reference LFM signal is delayed by the value of the target range to obtain $sr_{N}\left(\eta \right)$.
3. Generating a normalized white Gaussian noise, $N_{i}\left(\eta \right)$, with zero mean and unit variance corresponding to SNR values of −30 to 30 dB.
4. $N_{i}\left(\eta \right)$ and $sr_{N}\left(\eta \right)$ are added to obtain $srn_{N}\left(\eta \right)$, which is the received signal at MF.
5. IFFT the output of MF, $Xr_{N}\left(\omega \right)$, to obtain $xr_{N}\left(m\right)$.
6. $Xr_{N}\left(\omega \right)$ is multiplied by the frequency response of HW, $H_{W}\left(\omega \right)$, to obtain $Y_{HF}\left(\omega \right)$. IFFT of $Y_{HF}\left(\omega \right)$ to obtain $y_{HF}\left(m\right)$.
7. $Xr_{N}\left(\omega \right)$ is multiplied by $H_{SRF}$ to obtain $Y_{SRF}\left(\omega \right)$. IFFT of $Y_{SRF}\left(\omega \right)$ to obtain $y_{SRF}\left(m\right)$.
8. Having different performance measures (PSLR, IRW, MLR, and ROC) for the output of three filters; $xr_{N}\left(m\right)$, $y_{HF}\left(m\right)$, and $y_{SRF}\left(m\right)$.
9. To obtain the ROC, use the smallest-of-cell-averaging (SOF-CA) constant false alarm rate (CFAR) detector, which can detect very close targets [55]. A false alarm probability, $P_{fa}$, of $10^{-6}$, is considered.
10. For every value of SNR, repeat the previous steps 200 times using a Monte–Carlo simulation. Then, the mean value for each of the four metrics is calculated.

The PSLR is given by [56]:

(12) $$\mathrm{PSLR}=20\;\;\log_{10}\left(\mathrm{Peak}\;\mathrm{value}\;\mathrm{of}\;\mathrm{mainlobe}\;/\;\mathrm{Peak}\;\mathrm{value}\;\mathrm{of}\;\mathrm{first}\;\mathrm{sidelobe}\right)$$

**Figure 12:**
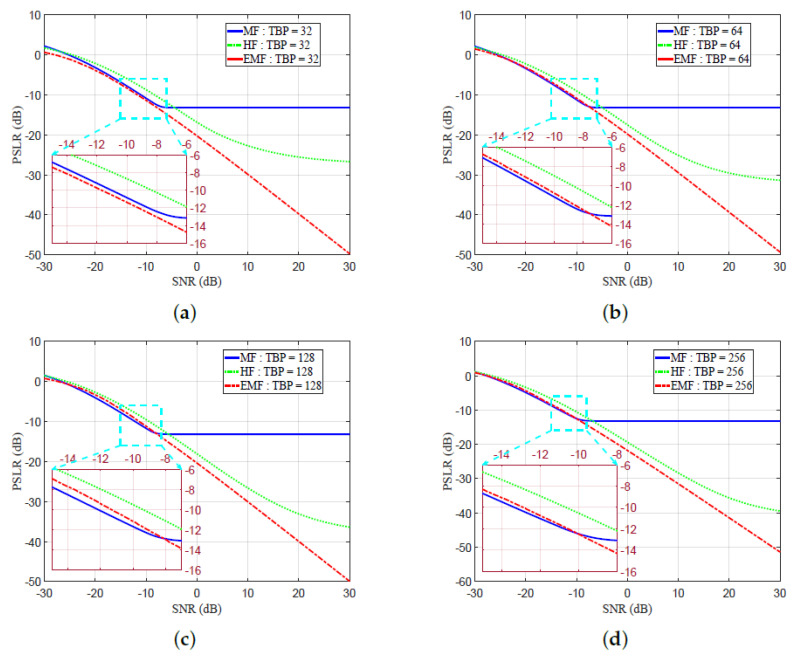
Peak sidelobe ratio (PSLR) versus SNR for MF, HF, and the proposed EMF: (**a**) TBP = 32, (**b**) TBP = 64, (**c**) TBP = 128, (**d**) TBP = 256.

Figure 12a–d shows the SNR versus PSLR, both measured in dB, for different values of TBP—32, 64, 128, and 256. For SNR low values, the HF, MF, and proposed EMF showed almost the same PSLR performance. In Figure 12a–d, EMF shows better PSLR measures than HF. When SNR increased over a certain value, EMF showed better PSLR measures than MF for all values of TBP, due to the effective sidelobe reduction by the EMF. A reduced PSLR indicates an enhanced detection performance.

The PSLR was enhanced with the proposed EMF compared to MF for all SNR values, for a TBP value of 32. The PSLR enhancements of the proposed EMF over MF started at SNR values of −7.5683, −8.1221, and −10.0833 dB for TBP values of 64, 128, and 256, respectively.

At SNR = 10 dB and TBP = 32, PSLR equaled −12.889, −22.712, and −29.857 dB for MF, HF, and the proposed EMF, respectively.

At SNR = 10 dB and TBP = 64, PSLR equaled −12.956, −25.1024, and −29.4391 dB for MF, HF, and proposed EMF, respectively.

At SNR = 10 dB, and TBP = 128, PSLR equaled −13.163, −26.6165, and −30.0499 dB for MF, HF, and the proposed EMF, respectively.

At SNR = 10 dB, and TBP = 256, PSLR equaled −13.192 dB, −28.3198 dB, and −31.4938 dB for MF, HF, and the proposed EMF, respectively.

Table 1 shows the IRW values (which represent the range resolutions in m) of HF, MF, and EMF for different values of TBPs and SNRs. The EMF provided almost the same range resolution as MF for different TBPs but high values of SNR. On the other hand, the EMF provided better range resolution compared to the HF, which severely degraded the IRW.

The MLR, measured in dB, is defined as the ratio of the mainlobe peak of the proposed EMF, or HF, to the mainlobe peak of MF. The MLR can be expressed as:

(13) $$\mathrm{MLR}=20\;\;\log_{10}\left(\frac{\mathrm{mainlobe}\;\mathrm{peak}\left(\mathrm{EMF}\;\mathrm{or}\;\mathrm{HF}\right)}{\mathrm{mainlobe}\;\;\mathrm{peak}\left(\mathrm{MF}\right)}\right)$$

Table 2 shows the MLRs of the proposed EMF and HF for different values of TBPs and SNRs.The proposed EMF maintained the mainlobe peak value with the minimum MLR, especially for high SNRs. For HF, the MLR converged to zero as SNR increased for any TBP value. Additionally, for a low SNR, the noise was the main cause of the increased value of the MLR.

**Figure 13:**
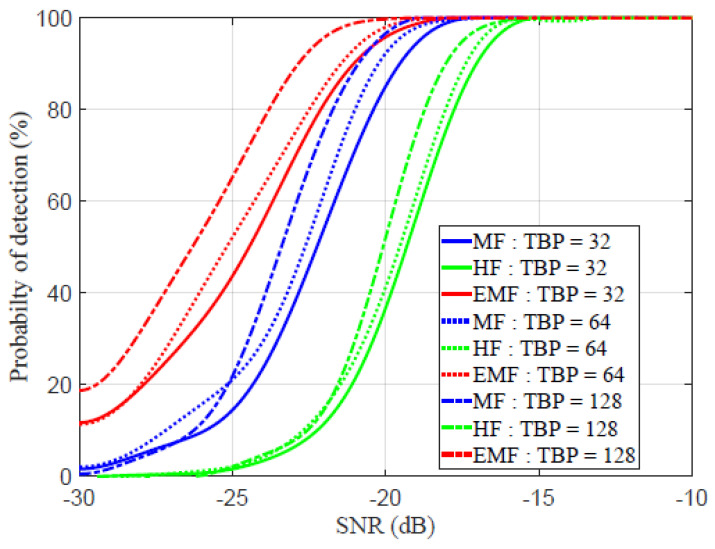
Receiver operational characteristic (ROC) for MF, HF, and the proposed EMF.

In Figure 13, the ROC is plotted against the SNR for different TBP values. The probability of detection is expressed by [57]:

(14) $$\mathrm{The}\;\mathrm{probability}\;\mathrm{of}\;\mathrm{detection}=\left(\frac{N_{tt}}{N_{fa}+N_{tt}+N_{mt}}\right)\times 100$$

where $N_{tt}$ is the number of targets that have been correctly identified, $N_{fa}$ is the number of falsely detected targets, and $N_{mt}$ is the number of targets that have been missed. The detection probability increased as SNR increased. For TBP=32, as detection probability approached 100%, the SNR of MF, HF, and EMF equaled −17.35, −15.2, and −17.9 dB, respectively. For TBP=64, as detection probability approached 100%, the SNR of MF, HF, and EMF equaled −16.95, −13.1, and −18.15 dB, respectively. For TBP=128, as the detection probability approached 100%, the SNRs of MF, HF, and EMF equaled −19, −15.45, and −19.35 dB, respectively. These values indicate that the detection capability of the EMF is better than those of the HF and MF, especially for low TBP values.

### 3.2. Ambiguity Function

In this subsection, the Doppler effect, which results due to the relative velocity between the transmitter and receiver, on the PSLR is investigated using the LFM radar ambiguity functions of the MF, HF, and EMF. For MF, the general form of the ambiguity function, $\chi_{\mathrm{MF}}\left(\tau ,\omega_{d}\right)$, is given by [29]:

(15) $$\begin{array}{c} \chi_{\mathrm{MF}}\left(\tau ,\omega_{d}\right)={\left|\int \left(S_{N}^{*}\left(\omega \right)\times S_{N}\left(\omega -\omega_{d}\right)\right)\times \exp\left(-j\omega \;\tau \right)\;\;d\omega \right|}^{2} \end{array}$$

where $S_{N}\left(\omega -\omega_{d}\right)=FFT\left[s_{N}\left(n\right)\times \exp\left(-j\omega_{d}\;\tau \right)\right]$, τ is the time delay corresponding to change in target range, and $\omega_{d}$ is the Doppler frequency shift.

For HF, the ambiguity function can be expressed as:

(16) $$\begin{array}{c} \chi_{\mathrm{HF}}\left(\tau ,\omega_{d}\right)={\left|\int \left(S_{N}^{*}\left(\omega \right)\times S_{N}\left(\omega -\omega_{d}\right)\times H_{W}\left(\omega \right)\right)\times \exp\left(-j\omega \;\tau \right)\;\;d\omega \right|}^{2} \end{array}$$

**Figure 14:**
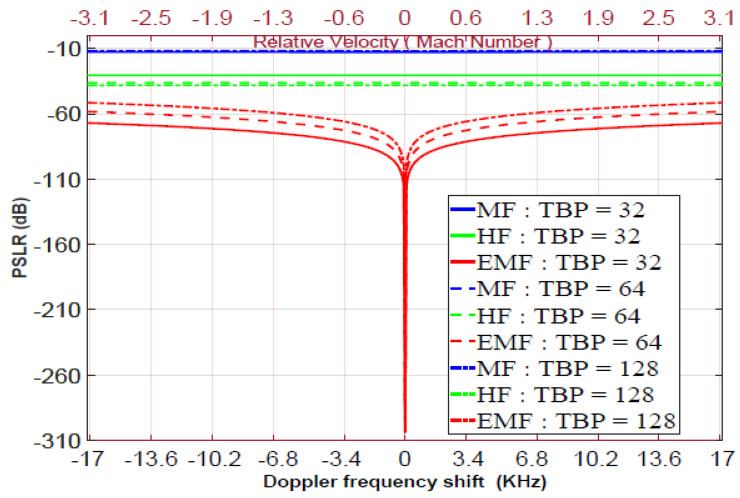
PSLR versus Doppler frequency for the LFM MF, HF, and proposed EMF.

For EMF, the ambiguity function can be expressed as:

(17) $$\begin{array}{c} \chi_{\mathrm{EMF}}\left(\tau ,\omega_{d}\right)={\left|\int \left(S_{N}^{*}\left(\omega \right)\times S_{N}\left(\omega -\omega_{d}\right)\times H_{SRF}\left(\omega \right)\right)\times \exp\left(-j\omega \;\tau \right)\;\;d\omega \right|}^{2} \end{array}$$

where $H_{SRF}\left(\omega \right)$ is the frequency response of the SRF, given by either Equation (10) or Equation (11). In Figure 14, the PSLR is plotted against the Doppler frequency $\omega_{d}$, in the range from −17 to 17 KHz. These $\omega_{d}$ values correspond to relative velocity from −3 to 3 Mach, at 2.4 GHz carrier frequency, $f_{c}$, (which will be used in the experimental work). The parameters of the pulsed LFM waveforms are: $\omega_{o}=0$; $B_{r}=90$ MHz; $f_{s}=120$ MHz; oversampling factor [54]; $OSF=f_{s}/B_{r}=1.33$; and TBPs of 32, 64, and 128. As shown in Figure 14, for TBP=32, EMF outperformed MF and HF for all values of $\omega_{d}$, especially at $\omega_{d}=0$. For $\omega_{d}=\pm \;17$ KHz and TBP=32, the PSLR of MF, HF, and EMF equals −12.823, −30.435, and −67.071 dB, respectively. For $\omega_{d}=\pm \;17$ KHz and TBP=64, the PSLRs of MF, HF, and EMF equal −12.193, −36.144, and −58.344 dB, respectively. For $\omega_{d}=\pm \;17$ KHz and TBP=128, the PSLRs of MF, HF, and EMF equal −11.815, −38.207, and −51.5205 dB, respectively. These values indicate that the detection capability of the proposed EMF is better than those of the HF and MF, especially for low TBP values.

The main drawback of EMF is that Doppler shifts affect its PSLR performance, unlike MF and HF, whose performances are almost unaffected by Doppler shifts. To practically implement the proposed SRF, it is necessary to reduce the Doppler effect shift by selecting lower operational carrier frequencies such as VHF, UHF, L-band, and S-band which are more suitable when detecting low-velocity targets such as ground vehicles, drones, and UAVs. To overcome this drawback, the Doppler effect shift can be compensated by replacing the value of $\omega_{o}$ in Equations (10) or (11) by $\omega_{o}+\omega_{d}$. Hence, the best performance for SRF was achieved at $\omega_{o}+\omega_{d}$ instead of $\omega_{o}$. The value of $\omega_{d}$ can be measured using conventional methods.

### 3.3. Comparison of EMF Performance with Sidelobe Reduction Techniques

In this subsection, the performance of the proposed EMF is compared to a modified MMF using APC and least squares for polyphase-code FM. The parameters given in [45] are reused here, considering a LFM waveform: first target, T1, with 80 dB SNR and range cell number 100; second target, T2, with 15 dB SNR and range cell number 95; total range samples of 200, $f_{s}=600$ MHz, $B_{r}=120$ MHz, and TBP=64.

**Figure 15:**
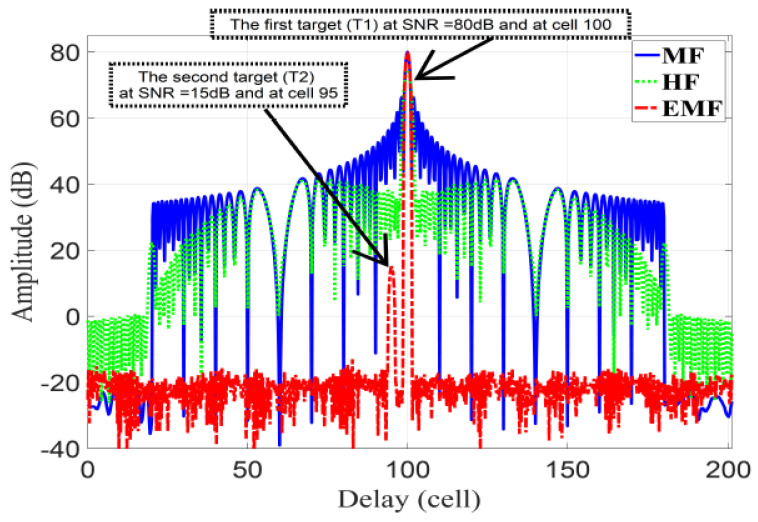
The amplitude response of the proposed EMF, MF, and HF.

Figure 15 represents the amplitude responses for MF, HF, and EMF. T2 is masked by the sidelobes of T1 for MF and HF, whereas EMF can discriminate T1 and T2 due to its effective reduction of the sidelobes of T1.

Table 3 lists the PSLR and IRW of T1 and T2, and the MLR of T1, for MF, HF, EMF, and the results of [45]. It can be seen in Table 3 that EMF achieved good performance in terms of the PSLR, while almost maintaining the IRW of MF. Additionally, the considerable reduction of MLR of T1 for EMF indicates the preservation of the mainlobe power. The missed data in Table 3, concerning T2 for MF and HF, was due to masking of T2 by the sidelobes of T1.

### 3.4. Performance Analysis Considering Multiple Targets

**Figure 16:**
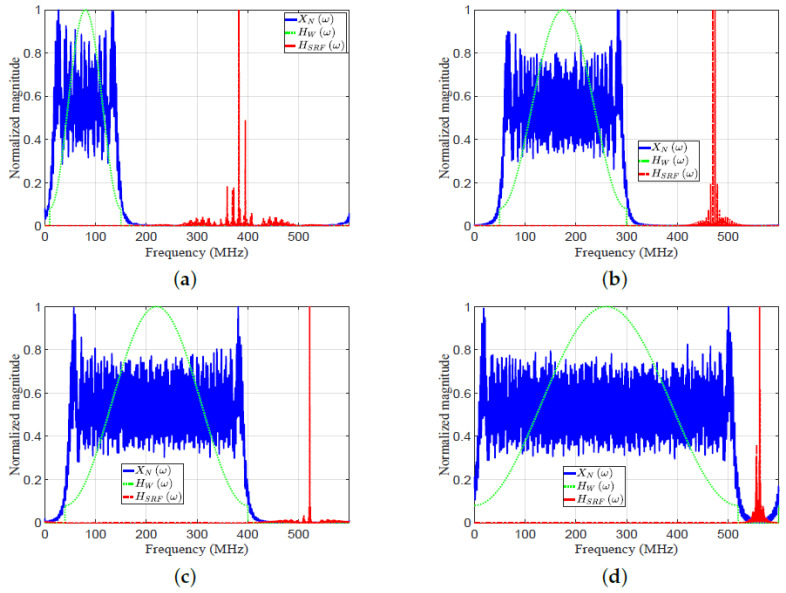
The frequency response $Xr_{N}\left(\omega \right)$, $H_{W}\left(\omega \right)$, and $H_{SRF}\left(\omega \right)$: (**a**) case 1, (**b**) case 2, (**c**) case 3, (**d**) case 4.

**Figure 17:**
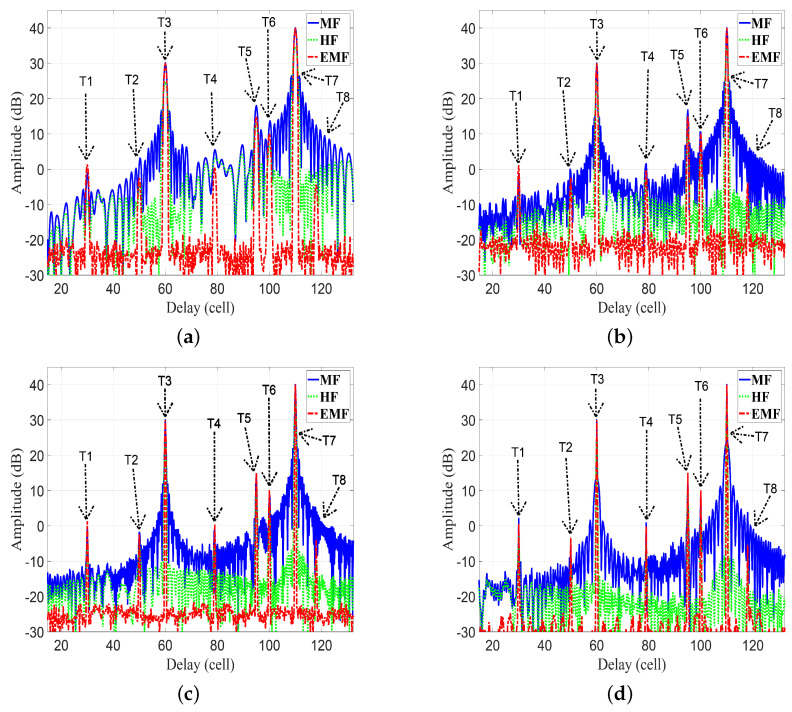
The amplitude responses of the MF, HF, and proposed EMF for different values of LFM waveform parameters: (**a**) case 1, (**b**) case 2, (**c**) case 3, (**d**) case 4.

In this subsection, the proposed EMF is compared with MF and HF for detecting multiple targets. Consider eight targets (T1 : T8) with the following parameters: Range cell numbers 30, 50, 60, 79, 95, 100, 110, and 118, respectively. SNRs of targets 1, −3, 30, 0, 15, 10, 40, and −5, respectively; $f_{s}=600$ MHz. Figure 16 plots the frequency response $Xr_{N}\left(\omega \right)$, $H_{W}\left(\omega \right)$, and $H_{SRF}\left(\omega \right)$. Figure 17 plots the amplitude responses versus range cells of HF, MF, and EMF.

Figure 16 and Figure 17 consider four cases of various parameters of the LFM waveform (*N*, position of zero frequency, OSF, $f_{0}$, $B_{r}$, and $N_{r}$). These cases were investigated to verify the efficiency of EMF for multi-targets detection with miscellaneous scenarios:

- For case 1 in Figure 16a and Figure 17a, the LFM waveform parameters are: N=236, zero-started frequency, OSF=4, $f_{o}=10$ MHz, $B_{r}=140$ MHz, and Nr=1229.
- For case 2 in Figure 16b and Figure 17b, the LFM waveform parameters are: N=415, zero-centered frequency, OSF=2, $f_{o}=175$ MHz, $B_{r}=250$ MHz, and Nr=1803.
- For case 3 in Figure 16c and Figure 17c, the LFM waveform parameters are: N=528, zero-started frequency, OSF=1.5, $f_{o}=40$ MHz, $B_{r}=360$ MHz, and Nr=2161.
- For case 4 in Figure 16d and Figure 17d, the LFM waveform parameters are: N=705, zero-centered frequency, OSF=1.1538, $f_{o}=260$ MHz, $B_{r}=520$ MHz, and Nr=2731.

As shown in Figure 17a–d, the performance of the designed EMF was better than the performances of MF and HF due to its effective reduction of the sidelobes for different values of LFM waveform’s parameters. All targets were detected successfully in the four cases by EMF. Target T8, of low SNR, was masked in the four cases due to its near location to T7, with high SNR. However, T8 was detected by the proposed EMF successfully due to its effective sidelobe reduction, while not being recognizable by MF and HF. Target T2 was masked in the first three cases, and T4 in the first two cases, due to the masking effect encountered by T3. In cases 3 and 4, the compression gain of MF increased due to increased N value. Consequently, T2 was detectable in case 4, and T4 was detectable in cases 3 and 4.

Table 4 lists the measured values of PSLR, IRW, and MLR, of T3 and T7, for MF, HF, and the proposed EMF, as shown in Figure 17a–d. The values of PSLR of T3 and T7 for EMF were greater than the values acquired by MF and HF, indicating a much better sidelobe reduction by EMF than by MF and HF. The values of IRW for EMF were almost the same as those of MF, whereas HF degraded the IRW values. This emphasizes the preservation of EMF to the targets’ range resolutions. The values of MLR for EMF were greater than those of HF, indicating that EMF keeps the peak value of the target’s mainlobe.

By applying the proposed EMF to the ultrasonic band, the performance of the proposed EMF is compared with MF for detecting multiple targets.

**Figure 18:**
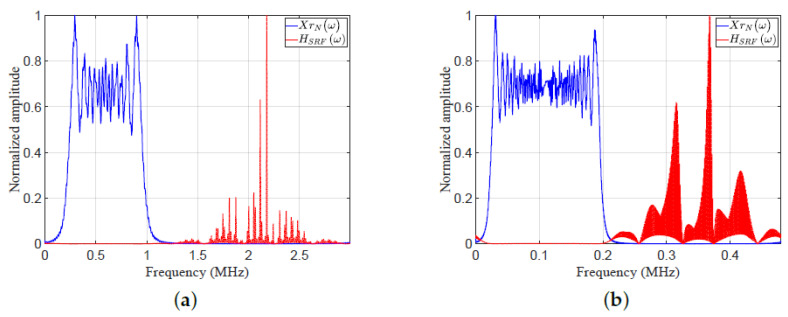
The frequency response $Xr_{N}\left(\omega \right)$, and $H_{SRF}\left(\omega \right)$: (**a**) an ultrasonic wave propagating through iron, and (**b**) an ultrasonic wave propagating through sea water.

**Figure 19:**
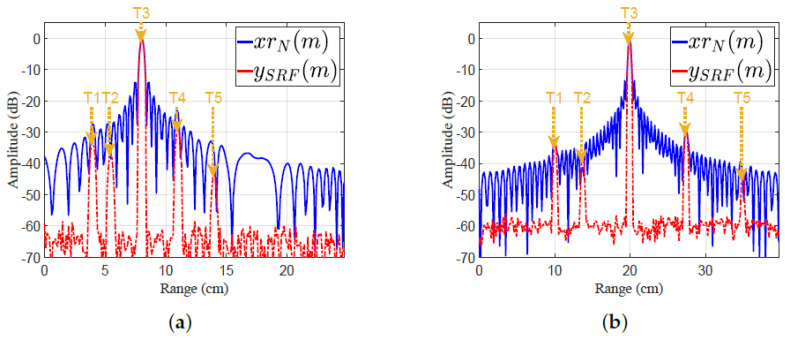
The amplitude responses of the MF and the proposed EMF for different values of LFM waveform parameters: (**a**) an ultrasonic wave propagating through iron, and (**b**) an ultrasonic wave propagating through sea water.

Consider five targets (T1:T5) with the following parameters: SNRs of targets 7, 3, 40, 11, and −3 respectively. Figure 18 shows a plots the frequency responses $Xr_{N}\left(\omega \right)$ and $H_{SRF}\left(\omega \right)$. Figure 19 shows plots of the amplitude response versus range cell for MF and EMF. Figure 18 and Figure 19 consider two cases of various parameters of LFM waveform (*N*, position of zero frequency, OSF, $f_{0}$, $B_{r}$, $f_{s}$, and $N_{r}$). These cases were investigated to verify the efficiency of EMF for multi-target detection with miscellaneous scenarios:

- Case 5, in Figure 18a and Figure 19a: five targets (T1:T5) have ranges of 4, 5.5, 8, 11, and 14 cm, respectively. The ultrasonic wave propagates through iron with speed ν=5960 m/sec.
  The LFM waveform parameters are: N=188, zero-started frequency, OSF=3, $f_{o}=200$ KHz, $B_{r}=800$ KHz, $f_{s}=3$ MHz, and Nr=424.
- Case 6, in Figure 18b and Figure 19b: five targets (T1:T5) have ranges of 10, 13.7, 20, 27.5, and 35 cm, respectively. The ultrasonic wave propagates through sea water with speed ν=1531 m/s.
  The LFM waveform parameters are: N=461, zero-centered frequency, OSF=2.4, $f_{o}=20$ KHz, $B_{r}=180$ KHz, $f_{s}=480$ KHz, and Nr=912.

As shown in Figure 19a,b, the performance of the proposed EMF was better than that of MF due to its effective reduction of the sidelobes for different values of LFM waveform parameters. All targets were detected successfully in the two cases by EMF. Targets T1, T2, T4, and T5 of low SNRs were masked in the two cases due to their nearness to T3, which had a high SNR. However, T1, T2, T4, and T5 were detected by the proposed EMF successfully due to its effective sidelobe reduction, but were not recognizable by MF, due to the masking effect encountered by T3.

Table 5 lists the measured values of PSLR, IRW, and MLR, of T3 and T5, for MF, and the proposed EMF, shown in Figure 19a,b. The values of PSLR of T3 and T5 for EMF are greater than the values acquired by MF, indicating a much better sidelobe reduction of EMF compared to MF. The values of IRW for EMF are almost the same as those of MF, which emphasizes the preservation by EMF of the targets’ range resolutions. The values of MLR for EMF indicate that EMF keeps the peak value of the target’s mainlobe.

From previous results, the proposed EMF can be applied to ultrasonic guided waves, active thermal non-destructive testing, and truncated-correlation photo-thermal coherence tomography.

## 4. Practical Proof Using SDR

The executed practical proof that validates the theory presented earlier in Section 2 is presented in this section. The pulsed LMF radar with the proposed EMF was implemented on Peripheral Component Interconnect (PCI) e**X**tensions for Instrumentation (PXI) system. PXI is a compact PC-based platform for automation systems and measurement built by National Instruments (NI). It has an Intel Core i7 processor within controller model PXIe-8135 and model PXIe-5644R which consists of a PXI Vector signal transceiver (VST). The VST is divided into a vector signal analyzer (Receiver; RX), vector signal generator (Transmitter; TX), and a field-programmable gate array (FPGA) real-time processing. The previous components were adjusted in chassis model PXIe-1082. An external function generator was used to adjust the proposed pulse repetition frequency (PRF) of the radar system. The experimental work was divided into two main phases: an indoor phase implemented in the laboratory, and an outdoor phase performed in an open field.

### 4.1. Experimental Work in the Laboratory

**Figure 20:**
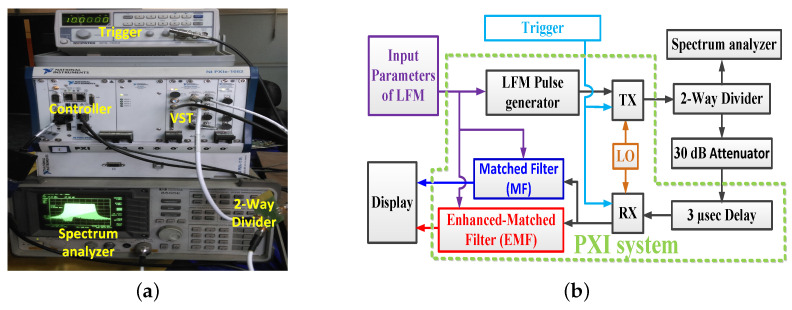
Experimental work in the laboratory: (**a**) Setup of the experiment. (**b**) Block diagram of experimental setup.

The experimental work in the laboratory was handled using the prescribed LMF radar system after programming the radar receiver with a MF and the proposed EMF. The experimental setup is shown in Figure 20a.

**Figure 21:**
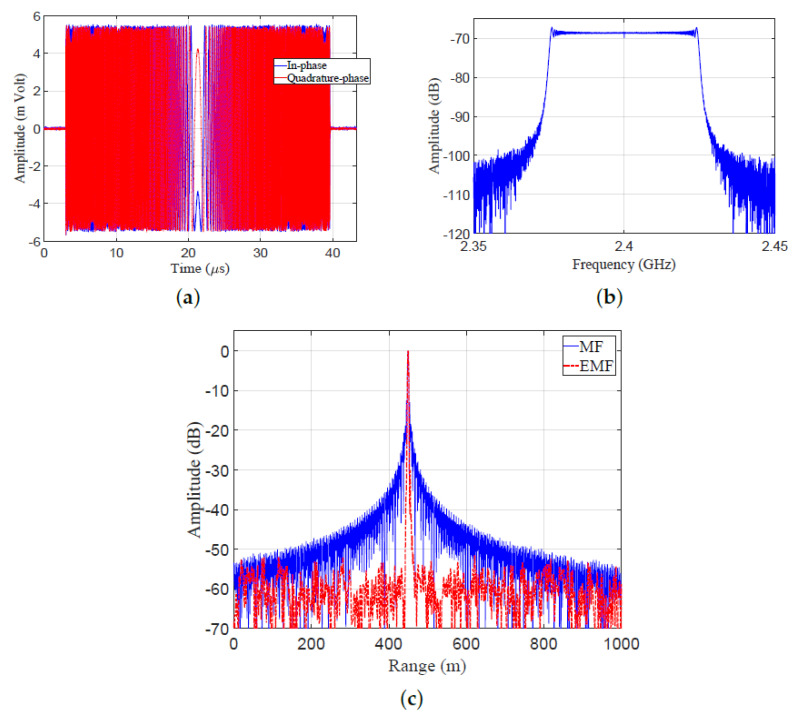
Results of the indoor experimental setup: (**a**) Received signal in the time domain. (**b**) Frequency spectrum of the received LFM signal. (**c**) Amplitude responses of MF and EMF.

The transmitted LFM signal was directed to the receiver by a closed loop between TX and RX, without using an antenna. Figure 20b shows a block diagram describing the experimental work: A zero-centered frequency LFM signal—$B_{r}=50$ Hz, $f_{c}=2.4$ GHz, $f_{s}=120$ MHz, N=4394, $N_{r}=5380$, PRF=10 KHz, and transmitted power =0 dBm—was produced by the LFM pulse generator. The frequency of the generated LFM signal was up-converted by a local oscillator (LO). The transmitted LFM signal was split using a 2-way RF splitter into two portions: The first one was shown on a spectrum analyzer to verify the band width and power of the LFM waveform. The second one was directed to RX, after being attenuated by a 30 dB attenuator, and then delayed by 3 μs. The received samples were down-converged at RX, and then processed by using MF and EMF. The results of the indoor experimental setup are shown in Figure 21. The in-phase and quadrature-phase of the received LFM signal in time domain are shown in Figure 21a. The frequency spectrum of the received LFM signal is shown in Figure 21b. The amplitude response of the LFM signal after being processed by both MF and EMF is shown in Figure 21c. It can be seen that the proposed EMF reduced sidelobes by 50 dB with respect to MF. These experimental results matche the theoretical results introduced earlier in Section 3.

### 4.2. Outdoor Experimental Work

**Figure 22:**
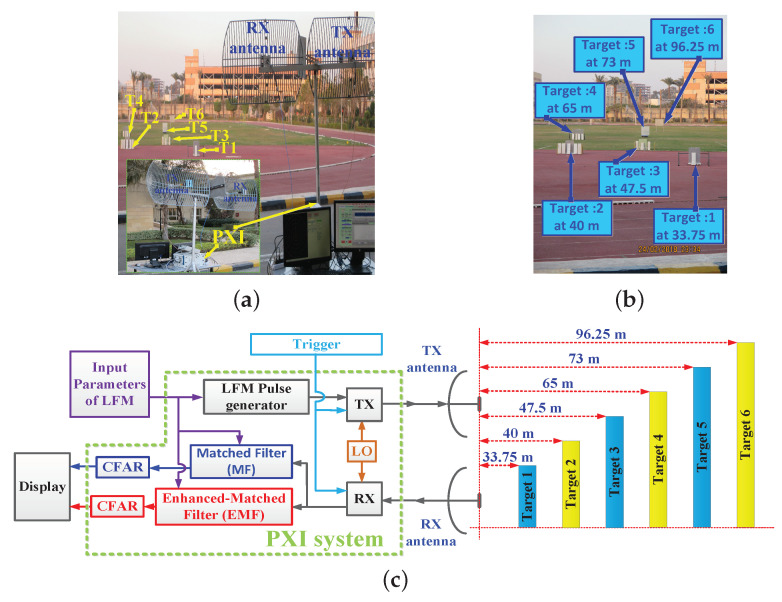
The open field experiment’s results: (**a**) Experimental setup. (**b**) Target locations. (**c**) Block diagram of the experiment setup.

The open field experimental work considered the detection of multiple targets. The experimental setup was the same as that used in the laboratory, but using two antennas as TX and RX instead of a single one; see Figure 22.

In this case, two antennas were used since the maximum output power from the PXI system was 0.1 watt, which is considered a very low power output for pulsed LFM radar. These antennas allowed the synchronous operation of TX and RX; hence echoes from targets within a short range could be received properly.

This guaranteed increasing the pulse width and consequently increasing the average power of the received signal, hence overcoming the dead zone problem which arises if a single antenna is used. Additionally, the usage of two antennas is better than using a circulator because the circulator attenuates the received signal [58] and improves the isolation level between TX and RX [59]. As shown in Figure 22a, two grid parabolic antennas, model TL-ANT2424B, operated at 2.4 GHz central frequency, with gain 24 dBi, were used. The power from TX was adjusted to 0 dBm. Six targets at different ranges were used, as shown in Figure 22b. The outdoor practical setup is represented in the block diagram shown in Figure 22c. CFAR was executed on the signals of both EMF and MF—$xr_{N}\left(m\right)$ and $y_{SRF}\left(m\right)$, respectively—to detect the six targets. The parameters of the transmitted LFM waveform, which was generated by the LFM pulse generator, included: zero-centered frequency LFM signal, $B_{r}=60$ Hz, $f_{c}=2.4$ GHz, $f_{s}=120$ MHz, N=4394, $N_{r}=4668$, PRF=1 KHz, and transmitted power =0 dBm.

**Figure 23:**
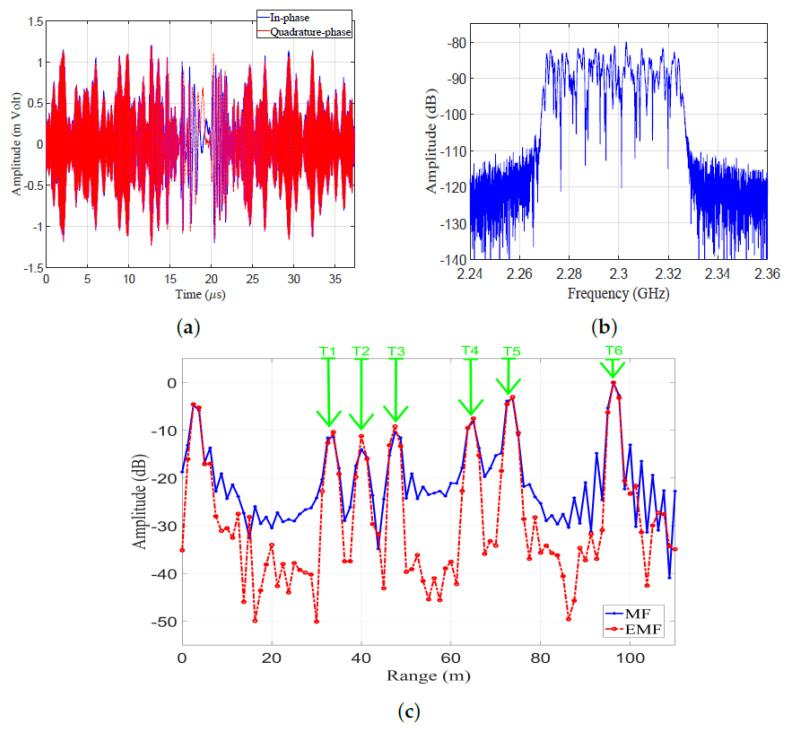
The open field experiment’s results: (**a**) Received signal in the time domain. (**b**) Frequency spectrum of the received LFM signal. (**c**) Amplitude responses of MF and proposed EMF.

Results of the outdoor experiment are shown in Figure 23. The in-phase and quadrature phase of the received LFM signal in the time domain are shown in Figure 23a, which represent the sums of reflections from the six targets and the reflections from clutters. The frequency spectrum of the received LFM signal is shown in Figure 23b. The amplitude responses of the LFM signal after being processed by EMF and MF are shown in Figure 23c. The outdoor experimental results match the theoretical results introduced in Section 3.

The IRW, PSLR, and MLR for every target in the outdoor experimental set-up were measured from Figure 23c, and are listed in Table 6. It can be noticed that the proposed EMF reduced the PSLRs and IRWs of the six targets, compared to MF. Additionally, increases in the peak values of the mainlobe were noticed when using EMF in contrast to MF.

**Figure 24:**
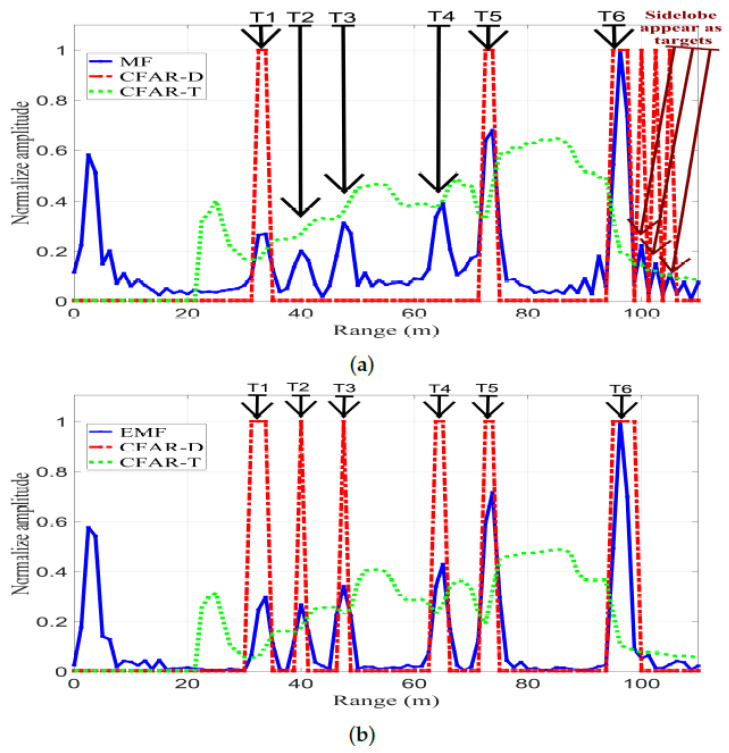
CFAR detection, with $P_{fa}=10^{-6}$, for MF and the proposed EMF: (**a**) MF. (**b**) Proposed EMF.

Figure 24 shows CFAR detection, with $P_{fa}=10^{-6}$, for the output signals of MF and EMF. CFAR-T and CFAR-D are CFAR threshold and detection, respectively. Any value of either MF or EMF above CFAR-T resulted in a CFAR-D value of 1, which corresponds to a detected target. Any value under CFAR-T resulted in a CFAR-D value of 0, which corresponds to an undetected target. Figure 24a shows CFAR detection for MF, where the CFAR-T level is high due to the existence of sidelobes; consequently, T2, T3, and T4 were not detected.

Additionally, the sidelobes corresponding to T6 appear as false targets due to their high RCS. Finally, only T1, T3, and T5 were detected.

Figure 24b shows CFAR detection for EMF: the CFAR-T level was reduced due to the efficient reduction of the sidelobes. Consequently, T1-T6 were all clearly detected without the presence of any false targets. Additionally, the sidelobes of T6 were considerably reduced by the proposed EMF, and hence the sidelobes corresponding to T6 do not appear as false targets. These results confirm practical efficiency of the designed and implemented EMF in sidelobe reduction and multi-target detection.

## 5. Conclusions

A new approach for LFM waveform sidelobe reduction in-range was introduced in this paper: an EMF combining SRF and MF which we implemented. One of the advantages of the new algorithm is that its generated SRF produces a frequency response from a derived mathematical model that depends on the LFM waveform parameters. Additionally, the designed EMF shows more enhanced PSLR measures than HF and common MF. The developed EMF was applied using a NI-PXI module and was assessed by evaluating the obtained MLR, PSLR, and IRW, and then comparing them to the corresponding ones of the common MF. The results showed that the applied EMF had a 50 dB sidelobe reduction compared to MF. Moreover, the EMF reduced IRWs and PSLRs more than MF. In addition, the mainlobe peak value was quite similar to that produced by MF and had a noticeable enhancement compared to the corresponding one when using HF. The proposed EMF can improve nondestructive testing and evaluation when using an LFM waveform due to its effective reduction of sidelobes and solving masking problems. In the paper, the findings of the theoretical analysis and the results from the experimental work carried out in a laboratory and in an open field matched and confirmed the remarkable efficiency of the proposed EMF in sidelobe reduction and multi-target detection.

## Figures and Tables

**Table 1:** Impulse response width (IRW) for HF, MF, and the proposed EMF.

TBP	Filter Type	−10 dB	0 dB	10 dB	20 dB
32	HF	2.5056	2.1269	1.9741	1.927
	MF	1.0027	0.83506	0.78469	0.77456
	EMF	1.1534	0.87025	0.79581	0.7745
64	HF	2.4596	2.0909	1.9542	1.9116
	MF	0.95456	0.80981	0.76706	0.75012
	EMF	1.1723	0.85037	0.77637	0.75544
128	HF	2.4441	2.0868	1.9548	1.9133
	MF	0.937	0.79969	0.75719	0.74975
	EMF	1.2516	0.84031	0.76406	0.74538

**Table 2:** Mainlobe loss ratio (MLR) for HF, and the proposed EMF.

TBP	Filter Type	−10 dB	0 dB	10 dB	20 dB
32	HF	−4.3013	−4.9058	−5.1326	−5.2087
	EMF	0.46276	0.16255	0.053266	0.017039
64	HF	−4.4288	−4.9825	−5.1881	−5.2568
	EMF	0.44535	0.15686	0.051422	0.016451
128	HF	−4.4959	−5.0393	−5.2406	−5.3077
	EMF	0.33908	0.11852	0.038769	0.012393

**Table 3:** PSLR comparison between EMF, MF, and HF in [45].

	EMF	MF	HF	[45]
PSLR T1 (dB)	−99.5948	−13.6184	−33.2513	−80
IRW T1 (m)	1.125	1.085	1.62	⋯
PSLR T2 (dB)	−32.5745	⋯	⋯	−20
IRW T2 (m)	1.0999	⋯	⋯	⋯
MLR T1(dB)	0.00026	⋯	−5.31091	⋯

**Table 4:** Measurements of PSLR, IRW, and MLR, of T3 and T7, for MF HF, and EMF, as shown in Figure 17.

	Target	Filter Type	PSLR (dB)	IRW (m)	MLR (dB)
Figure 17a	T3	MF	−13.451	3.71	⋯
		HF	−31.835	5.55	−5.3521
		EHF	−51.195	3.69	−0.062637
	T7	MF	−13.633	3.71	⋯
		HF	−37.415	5.55	−5.3267
		EHF	−61.104	3.7	−0.015993
Figure 17b	T3	MF	−17.742	2.08	⋯
		HF	−35.825	2.98	−5.3315
		EHF	−48.673	2.07	−0.023585
	T7	MF	−17.914	2.08	⋯
		HF	−44.074	2.96	−5.3565
		EHF	−56.545	2.07	−0.0078977
Figure 17c	T3	MF	−21.023	1.02	⋯
		HF	−34.92	2.12	−5.3468
		EHF	−53.362	1.02	0.0014252
	T7	MF	−21.191	1.02	⋯
		HF	−41.352	2.12	−5.3525
		EHF	−62.887	1.02	0.0050595
Figure 17d	T3	MF	−28.408	0.38	⋯
		HF	−40.686	1.11	−5.3567
		EHF	−59.512	0.38	0.0085093
	T7	MF	−28.65	0.38	⋯
		HF	−44.026	1.12	−5.3529
		EHF	−67.991	0.38	0.0037488

**Table 5:** Measurements of PSLR, IRW, and MLR, of T3 and T5, for MF and EMF, as shown in Figure 19.

	Target	Filter Type	PSLR (dB)	IRW (cm)	MLR (dB)
Figure 19a	T3	MF	−14.06	3.13	⋯
		EHF	−61.719	3.1	−0.0095694
	T5	MF	⋯	⋯	⋯
		EHF	−18.338	2.41	⋯
Figure 19b	T3	MF	−13.48	2.22	⋯
		EHF	−56.796	2.22	0.0071165
	T5	MF	⋯	⋯	⋯
		EHF	−15.618	1.99	⋯

**Table 6:** Measurements of IRW, PSLR, and MLR for the targets of the outdoor experiment.

Target	IRW (m)		PSLR (dB)		MLR (dB)
	MF	EMF	MF	EMF	EMF
T1	2.2375	1.7875	−17.5351	−27.0832	1.0484
T2	2.5625	1.2375	−14.9137	−26.2448	2.8314
T3	2.2125	1.8625	13.8235	−30.5081	1.2425
T4	2.200	1.8375	−11.5365	−28.375	0.66079
T5	2.025	1.8875	−16.3496	−31.1558	0.34026
T6	1.9875	1.775	−13.0407	−23.3083	0.0040

**Figure A1:**
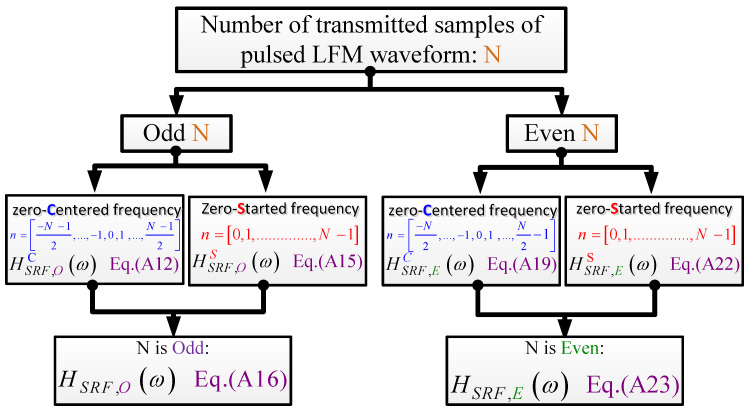
Generation of the frequency response of SRF for even and odd values of *N*.

## Data Availability

Not applicable.

## Appendix A. Derivation of the Frequency Response of the SRF; $H_{SRF}\left(\omega \right)$

This derivation is performed according to the number of samples, within the waveform, *N*, and the parameters of LFM waveform including $f_{s}$ and $B_{r}$. The desired output signal is the peak value of $x_{N}\left(m\right)$ and the rest of samples are zeroed [47]. Hence, the resulting bandwidth of the desired output is widened. Consequently, the SNR is reduced. In order to increase the SNR in the EMF, it is mandatory to reduce the bandwidth of the desired output. This can be achieved by modifying the desired output signal in the time domain, $d_{SRF,N}\left(m\right)$, which will be composed of the samples in the mainlobe of $x_{N}\left(m\right)$, and by zeroing the rest of samples outside the mainlobe. An empirical formula of the number of samples in the mainlobe, $N_{m}$, based on selecting a fixed value of $f_{s}$ and varying the values of $B_{r}$, is expressed as:

(A1) $$N_{m}=1+\;2\;N_{u}$$

where $N_{u}=\left\lfloor f_{s}/B_{r}.\right\rfloor$.

For a pulsed LFM waveform with $f_{s}=120$ MHz and $B_{r}=50$ MHz, $N_{m}=5$: this value of $N_{m}$ will be used to verify the derivation of the frequency response of the SRF.

### Appendix A.1. Derivation of $H_{SRF}\left(\omega \right)$:

In this subsection, $H_{SRF}\left(\omega \right)$ will be derived considering the discrete single-pulse LFM signal s(n) in Equation (1) for even and odd values of *N*. Accordingly, $H_{SRF}\left(\omega \right)$ is defined as:

(A2) $$H_{SRF}\left(\omega \right)=\;\;\;\left\{\begin{array}{c} H_{SRF,O}\left(\omega \right)\;\;\;\;\;\;\mathrm{for}\;\mathrm{odd}\;N\\ H_{SRF,E}\left(\omega \right)\;\;\;\;\;\;\mathrm{for}\;\mathrm{even}\;N \end{array}\right.\;$$

The methodology of deriving $H_{SRF,O}\left(\omega \right)$ and $H_{SRF,E}\left(\omega \right)$ can be summarized as follows:

1. Autocorrelation for the discrete single-pulse LFM signal, $s_{N}\left(n\right)$, to obtain $x_{N}\left(m\right)$.
2. FFT of $x_{N}\left(m\right)$ to get $X_{N}\left(\omega \right)$, which is the input signal of the SRF.
3. The desired output signal, $d_{SRF,N}\left(m\right)$, is composed of the samples in the mainlobe of $x_{N}\left(m\right)$, while zeroing the rest of samples outside the mainlobe.
4. FFT of $d_{SRF,N}\left(m\right)$ to get $D_{SRF,N}\left(\omega \right)$.
5. The frequency response of the SRF is $H_{SRF,N}\left(\omega \right)=D_{SRF,N}\left(\omega \right)/X_{N}\left(\omega \right)$.
6. Manipulating $H_{SRF,N}\left(\omega \right)$ to obtain a compact form.
7. For odd N of an LFM waveform with zero-centered frequency and zero-started frequency, $H_{SRF,O}^{C}\left(\omega \right)$ and $H_{SRF,O}^{S}\left(\omega \right)$ are generated, respectively, which are then combined to get $H_{SRF,O}\left(\omega \right)$. as shown in Figure A1.
8. For even N of an LFM waveform with zero-centered frequency and zero-started frequency, $H_{SRF,E}^{C}\left(\omega \right)$ and $H_{SRF,E}^{S}\left(\omega \right)$ are generated, respectively, which are then combined to get $H_{SRF,E}\left(\omega \right)$. as shown in Figure A1.

#### Appendix A.1.1. Derivation of $H_{SRF,O}\left(\omega \right)$ for odd *N*:

$H_{SRF,O}\left(\omega \right)$ is obtained by considering the frequency responses of the SRF for an LFM waveform with zero-centered frequency and zero-started frequency, $H_{SRF,O}^{C}\left(\omega \right)$ and $H_{SRF,O}^{S}\left(\omega \right)$, respectively.

For odd N and zero-centered frequency; $H_{SRF,11}^{C}\left(\omega \right)$:

$H_{SRF,11}^{C}\left(\omega \right)$, for N = 11, and $H_{SRF,13}^{C}\left(\omega \right)$, for N = 13, are used to get a general formula for $H_{SRF,O}^{C}\left(\omega \right)$.

$H_{SRF,11}^{C}\left(\omega \right)$ and $H_{SRF,13}^{C}\left(\omega \right)$: Consider the general form of a transmitted discrete single-pulsed LFM waveform $s_{N}\left(n\right)$, given in Equation (1).

For N=11 and zero-centered frequency,

(A3) $$\begin{array}{cc} s_{11}\left(n\right) & =[e^{j(25\pi k-5\omega_{o})},e^{j(16\pi k-4\omega_{o})},e^{j(9\pi k-3\omega_{o})},e^{j(4\pi k-2\omega_{o})},e^{j(\pi k-\omega_{o})},1,\\ \; & \;\;\;\;\;\;\;\;\;\;\;\;\;\;\;\;\;\;\;\;\;e^{j(\pi k+\omega_{o})},e^{j(4\pi k+2\omega_{o})},e^{j(9\pi k+3\omega_{o})},e^{j(16\pi k+4\omega_{o})},e^{j(25\pi k+5\omega_{o})}] \end{array}$$

where n={−5,−4,−3,−2,−1,0,1,2,3,4,5}. The autocorrelation function $x_{11}\left(m\right)$, for $s_{11}\left(n\right)$, considers a linear correlation whose length is 2N−1=21, and it can be written as:

(A4) $$x_{11}\left(m\right)=\sum_{n=-\infty }^{\infty }s_{11}^{*}\left(n\right)s_{11}\left(n+m-N\right)\;\;\;,\;m=1,\;2,\;3,.....,2N-2,2N-1$$

Hence, $x_{11}\left(m\right)$ has 21 $\left\{q_{1},q_{1},q_{2},\cdots ,q_{21}\right\}$ which are given by:

$$\begin{array}{cc} q_{1} & =e^{j(25k\pi -5\omega_{o})}e^{-j(25k\pi +5\omega_{o})}\;,\\ q_{2} & =e^{j(25k\pi -5\omega_{o})}e^{-j(16k\pi +4\omega_{o})}+e^{j(16k\pi -4\omega_{o})}e^{-j(25k\pi +5\omega_{o})}\;,\\ q_{3} & =e^{j(25k\pi -5\omega_{o})}e^{-j(9k\pi +3\omega_{o})}+e^{j(16k\pi -4\omega_{o})}e^{-j(16k\pi +4\omega_{o})}+e^{j(9k\pi -3\omega_{o})}e^{-j(25k\pi +5\omega_{o})}\;,\\ q_{4} & =e^{j(25k\pi -5\omega_{o})}e^{-j(4k\pi +2\omega_{o})}+e^{j(16k\pi -4\omega_{o})}e^{-j(9k\pi +3\omega_{o})}+e^{j(9k\pi -3\omega_{o})}e^{-j(16k\pi +4\omega_{o})}\\ \; & \;\;\;+\;e^{j(4k\pi -2\omega_{o})}e^{-j(25k\pi +5\omega_{o})}\;,\\ q_{5} & =e^{j(25\pi k-5\omega_{o})}e^{-j(\pi k+\omega_{o})}+e^{j(16\pi k-4\omega_{o})}e^{-j(4\pi k+2\omega_{o})}+e^{j(9\pi k-3\omega_{o})}e^{-j(9\pi k+3\omega_{o})}\\ \; & \;\;\;+e^{j(4\pi k-2\omega_{o})}e^{-j(16\pi k-4\omega_{o})}+e^{j(\pi k-\omega_{o})}e^{-j(25\pi k-5\omega_{o})}\;,\\ q_{6} & =e^{j(25\pi k-5\omega_{o})}+e^{-j(25\pi k+5\omega_{o})}+e^{j(16\pi k-4\omega_{o})}e^{-j(\pi k+\omega_{o})}+e^{j(9\pi k-3\omega_{o})}e^{-j(4\pi k+2\omega_{o})}\\ \; & \;\;\;+e^{j(4\pi k-2\omega_{o})}e^{-j(9\pi k+3\omega_{o})}+e^{j(\pi k-\omega_{o})}e^{-j(16\pi k+4\omega_{o})}\;,\\ q_{7} & =e^{j(25\pi k-5\omega_{o})}e^{-j(\pi k-\omega_{o})}+e^{j(16\pi k-4\omega_{o})}+e^{-j(16\pi k+4\omega_{o})}+e^{j(9\pi k-3\omega_{o})}e^{-j(\pi k+\omega_{o})}\\ \; & \;\;\;+e^{j(4\pi k-2\omega_{o})}e^{-j(4\pi k+2\omega_{o})}+e^{j(\pi k-\omega_{o})}e^{-j(9\pi k+3\omega_{o})}+e^{j(\pi k+\omega_{o})}e^{-j(25\pi k+5\omega_{o})}\;,\\ q_{8} & =e^{j(25\pi k-5\omega_{o})}e^{-j(4\pi k-2\omega_{o})}+e^{j(16\pi k-4\omega_{o})}e^{-j(\pi k-\omega_{o})}+e^{j(9\pi k-3\omega_{o})}+e^{-j(9\pi k+3\omega_{o})}\\ \; & \;\;\;+e^{j(4\pi k-2\omega_{o})}e^{-j(\pi k+\omega_{o})}+e^{j(\pi k-\omega_{o})}e^{-j(4\pi k+2\omega_{o})}+e^{j(\pi k+\omega_{o})}e^{-j(16\pi k+4\omega_{o})}\\ \; & \;\;\;+e^{j(4\pi k+2\omega_{o})}e^{-j(25\pi k+5\omega_{o})}\;,\\ q_{9} & =e^{j(25\pi k-5\omega_{o})}e^{-j(9\pi k-3\omega_{o})}+e^{j(16\pi k-4\omega_{o})}e^{-j(4\pi k-2\omega_{o})}+e^{j(9\pi k-3\omega_{o})}e^{-j(\pi k-\omega_{o})}|\\ \; & \;\;\;+e^{j(4\pi k-2\omega_{o})}+e^{-j(4\pi k+2\omega_{o})}+e^{j(\pi k-\omega_{o})}e^{-j(\pi k+\omega_{o})}+e^{j(\pi k+\omega_{o})}e^{-j(9\pi k+3\omega_{o})}\\ \; & \;\;\;\;\;\;+e^{j(4\pi k+2\omega_{o})}e^{-j(16\pi k+4\omega_{o})}+e^{j(9\pi k+3\omega_{o})}e^{-j(25\pi k+5\omega_{o})}\;,\\ q_{10} & =e^{j(25\pi k-5\omega_{o})}e^{-j(16\pi k-4\omega_{o})}+e^{j(16\pi k-4\omega_{o})}e^{-j(9\pi k-3\omega_{o})}+e^{j(9\pi k-3\omega_{o})}e^{-j(4\pi k-2\omega_{o})}\\ \; & \;\;\;+e^{j(4\pi k-2\omega_{o})}e^{-j(\pi k-\omega_{o})}+e^{j(\pi k-\omega_{o})}+e^{-j(\pi k+\omega_{o})}+e^{j(\pi k+\omega_{o})}e^{-j(4\pi k+2\omega_{o})}\\ \; & \;\;\;\;\;\;+e^{j(4\pi k+2\omega_{o})}e^{-j(9\pi k+3\omega_{o})}+e^{j(9\pi k+3\omega_{o})}e^{-j(16\pi k+4\omega_{o})}+e^{j(16\pi k+4\omega_{o})}e^{-j(25\pi k+5\omega_{o})}\;,\;\mathrm{and}\\ q_{11} & =11\;\;. \end{array}$$

whereas $q_{12}$ to $q_{21}$ are expressed as: $q_{11+l}=q_{11-l}^{*}$, l={1,2,⋯,10}.

Next, apply FFT on $x_{11}\left(m\right)$ to obtain $X_{11}\left(\omega \right)$:

(A5) $$X_{11}\left(\omega \right)=\left(\sum_{m=0}^{20}x_{11}\left(m\right)e^{-j\frac{2\pi m\omega }{21}}\right)e^{j10\omega }$$

For simplicity, Equation (A5) can be expressed as:

(A6) $$X_{11}\left(\omega \right)=11+\sum_{\lambda =1}^{10}A_{\lambda }$$

where $A_{\lambda }=q_{11-\lambda }\;e^{j\lambda \omega }+q_{11+\lambda }\;e^{-j\lambda \omega }$.

The desired output in time domain for N=11, $d_{SRF,11}^{C}\left(m\right)$, which is composed of the samples in the mainlobe, $x_{11}\left(m\right)$, while zeroing the rest of samples outside the mainlobe, for $N_{m}=5$, can be written as:

(A7) $$d_{SRF,11}^{C}\left(m\right)=\left\{0,0,0,0,0,0,0,0,\;q_{9}\;,\;q_{10}\;,\;11\;,\;q_{12}\;,\;q_{13}\;,0,0,0,0,0,0,0,0\right\}$$

FFT of $d_{SRF,11}^{C}\left(m\right)$ to get $D_{SRF,11}^{C}\left(\omega \right)$:

(A8) $$\begin{array}{cc} D_{SRF,11}^{C}\left(\omega \right) & =\left(\sum_{m=0}^{20}dm_{SRF,11}^{C}\left(m\right)e^{-j\frac{2\pi m\omega }{21}}\right)e^{j10\omega }\\ \; & =11+q_{10}\;e^{j\omega }+q_{12}\;e^{-j\omega }+q_{9}\;e^{j2\omega }+q_{13}\;e^{-j2\omega }\\ \; & =11+\sum_{\lambda =1}^{2}A_{\lambda } \end{array}$$

Since

(A9) $$H_{SRF,11}^{C}\left(\omega \right)=D_{SRF,11}^{C}\left(\omega \right)/X_{11}\left(\omega \right)$$

Then, from Equations (A6) and (A8), and after manipulations, a simplified formula for $H_{SRF,11}^{C}\left(\omega \right)$ can be written as:

(A10) $$H_{SRF,11}^{C}\left(\omega \right)=\frac{11+\sum_{\lambda =1}^{2}Bc_{\lambda }^{11}\left(\omega \right)}{11+\sum_{\lambda =1}^{10}Bc_{\lambda }^{11}\left(\omega \right)}$$

where

$$\begin{array}{cc} \; & Bc_{1}^{11}=4\left[\cos(k\pi )+\cos(3k\pi )+\cos(5k\pi )+\cos(7k\pi )+\cos(9k\pi )\right]\cos\left(\;\;(\omega -\omega_{o})\right)\;,\;\;\;\;\;\;\;\;\;\;\;\;\;\;\;\;\;\;\;\;\;\;\;\;\;\;\;\;\;\;\;\;\;\;\;\;\\ \; & Bc_{2}^{11}=\left[2+4\cos(4k\pi )+4\cos(8k\pi )+4\cos(12k\pi )+4\cos(16k\pi )\right]\cos\left(2(\omega -\omega_{o})\right)\;,\\ \; & Bc_{3}^{11}=4\left[\cos(3k\pi )+\cos(9k\pi )+\cos(15k\pi )+\cos(21k\pi )\right]\cos\left(3(\omega -\omega_{o})\right)\;,\\ \; & Bc_{4}^{11}=\left[2+4\cos(8k\pi )+4\cos(16k\pi )+4\cos(24k\pi )\right]\cos\left(4(\omega -\omega_{o})\right)\;,\\ \; & Bc_{5}^{11}=4\left[\cos(5k\pi )+\cos(15k\pi )+\cos(25k\pi )\right]\cos\left(5(\omega -\omega_{o})\right)\;,\\ \; & Bc_{6}^{11}=\left[2+4\cos(14k\pi )+4\cos(24k\pi )\right]\cos\left(6(\omega -\omega_{o})\right)\;,\\ \; & Bc_{7}^{11}=4\left[\cos(7k\pi )+\cos(21k\pi )\right]\cos\left(7(\omega -\omega_{o})\right)\;,\\ \; & Bc_{8}^{11}=\left[2+4\cos(16k\pi )\right]\cos\left(8(\omega -\omega_{o})\right)\;,\\ \; & Bc_{9}^{11}=4\left[\cos(9k\pi )\right]\cos\left(9(\omega -\omega_{o})\right)\;,\;\mathrm{and}\\ \; & Bc_{10}^{11}=2\cos\left(10(\omega -\omega_{o})\right). \end{array}$$

For N=13 and zero-centered frequency, and by following the same procedures used for the derivation of $H_{SRF,11}^{C}\left(\omega \right)$, $H_{SRF,13}^{C}\left(\omega \right)$ can be written as:

(A11) $$H_{SRF,13}^{C}\left(\omega \right)=\frac{13+\sum_{\lambda =1}^{2}Bc_{\lambda }^{13}\left(\omega \right)}{13+\sum_{\lambda =1}^{12}Bc_{\lambda }^{13}\left(\omega \right)}$$

where

$$\begin{array}{cc} \; & Bc_{1}^{13}=4\left[\cos(k\pi )+\cos(3k\pi )+\cos(5k\pi )+\cos(7k\pi )+\cos(9k\pi )+\cos(11k\pi )\right]\;\;\;\;\;\;\;\;\;\;\;\;\;\;\;\;\\ \; & \;\;\;\;\;\;\;\;\;\;\;\;\;\;\;\;\;\;\;\;\;\;\;\;\;\;\;\;\;\;\;\;\;\;\;\;\;\;\;\;\;\;\;\;\;\;\;\;\;\;\;\;\;\;\;\;\;\;\;\;\;\;\;\;\;\;\;\;\;\;\;\;\;\;\;\;\;\;\;\;\;\;\;\;\;\;\;\;\;\;\;\;\;\;\;\cos\left(\;\;(\omega -\omega_{o})\right)\;,\\ \; & Bc_{2}^{13}=\left[2+4\cos(4k\pi )+4\cos(8k\pi )+4\cos(12k\pi )+4\cos(16k\pi )+4\cos(20k\pi )\right]\\ \; & \;\;\;\;\;\;\;\;\;\;\;\;\;\;\;\;\;\;\;\;\;\;\;\;\;\;\;\;\;\;\;\;\;\;\;\;\;\;\;\;\;\;\;\;\;\;\;\;\;\;\;\;\;\;\;\;\;\;\;\;\;\;\;\;\;\;\;\;\;\;\;\;\;\;\;\;\;\;\;\;\;\;\;\;\;\;\;\;\;\;\;\;\;\;\;\cos\left(2(\omega -\omega_{o})\right)\;,\\ \; & Bc_{3}^{13}=4\left[\cos(3k\pi )+\cos(9k\pi )+\cos(15k\pi )+\cos(21k\pi )+\cos(27k\pi )\right]\cos\left(3(\omega -\omega_{o})\right)\;,\\ \; & Bc_{4}^{13}=\left[2+4\cos(8k\pi )+4\cos(16k\pi )+4\cos(24k\pi )+4\cos(32k\pi )\right]\cos\left(4(\omega -\omega_{o})\right)\;,\\ \; & Bc_{5}^{13}=4\left[\cos(5k\pi )+\cos(15k\pi )+\cos(25k\pi )+\cos(35k\pi )\right]\cos\left(5(\omega -\omega_{o})\right)\;,\\ \; & Bc_{6}^{13}=\left[2+4\cos(12k\pi )+4\cos(24k\pi )+4\cos(36k\pi )\right]\cos\left(6(\omega -\omega_{o})\right)\;,\\ \; & Bc_{7}^{13}=4\left[\cos(7k\pi )+\cos(21k\pi )+\cos(35k\pi )\right]\cos\left(7(\omega -\omega_{o})\right)\;,\\ \; & Bc_{8}^{13}=\left[2+4\cos(16k\pi )+4\cos(32k\pi )\right]\cos\left(8(\omega -\omega_{o})\right)\;,\\ \; & Bc_{9}^{13}=4\left[\cos(9k\pi )+\cos(27k\pi )\right]\cos\left(9(\omega -\omega_{o})\right)\;,\\ \; & Bc_{10}^{13}=\left[2+4\cos(20k\pi )\right]\cos\left(10(\omega -\omega_{o})\right)\;,\\ \; & Bc_{11}^{13}=4\left[\cos(11k\pi )\right]\cos\left(11(\omega -\omega_{o})\right)\;,\;\mathrm{and}\\ \; & Bc_{12}^{13}=2\cos\left(12(\omega -\omega_{o})\right)\;. \end{array}$$

**The general formula of**$H_{SRF,O}^{C}\left(\omega \right)$: A general formula of $H_{SRF,O}^{C}\left(\omega \right)$ is obtained by combining $H_{SRF,11}^{C}\left(\omega \right)$ and $H_{SRF,13}^{C}\left(\omega \right)$, given by Equations (A10) and (A11), respectively. After manipulations, $H_{SRF,O}^{C}\left(\omega \right)$ can be expressed as:

(A12) $$H_{SRF,O}^{C}\left(\omega \right)=\frac{H_{no}^{C}\left(\omega \right)}{H_{do}^{C}\left(\omega \right)}$$

where

$$\begin{array}{cc} \; & H_{no}^{C}\left(\omega \right)=N+\sum_{a=1}^{\frac{N_{u}}{2}+1}{FO}_{N}^{C}+\sum_{a=1}^{\frac{N_{u}}{2}}{FE}_{N}^{C}\;\;,\\ \; & H_{do}^{C}\left(\omega \right)=N+2\cos\left(\left(N-1\right)\;\Omega_{CNo}\right)+\sum_{a=1}^{\frac{N-1}{2}}{FO}_{N}^{C}+\sum_{a=1}^{\frac{N-1}{2}\;-\;1}{FE}_{N}^{C}\;\;,\\ \; & {FO}_{N}^{C}=4\left(\sum_{b=1}^{\frac{N+1}{2}-a}O_{N}\right)\cos\left(\left(2a-1\right)\Omega_{CNo}\right)\;,\;\;{FE}_{N}^{C}=2\left(\sum_{b=1}^{\frac{N-1}{2}\;-a}E_{N}+1\right)\cos\left(2\;a\;\Omega_{CNo}\right)\;,\\ \; & \Omega_{CNo}=\omega -\omega_{o},\;\;\;O_{N}=\cos\left(\left(2a-1\right)\left(2b-1\right)\pi \;k\right),\;\;\;E_{N}=2\cos\left(4\;\pi \;a\;b\;k\right),\;\;\;\mathrm{and}\;\;\;\;\;\;\;\;\;\;\;\;\;\;\;\;\;\;\;\;\;\;\;\;\;\;\;\;\; \end{array}$$

For odd *N* and zero-started frequency; $H_{SRF,O}^{C}\left(\omega \right)$

Consider two values of *N*, 11 and 13, to get $H_{SRF,11}^{S}\left(\omega \right)$ and $H_{SRF,13}^{S}\left(\omega \right)$, respectively, which will be used to deduce a general form of $H_{SRF,O}^{S}\left(\omega \right)$.

**Derivation of**$H_{SRF,11}^{S}\left(\omega \right)\;\mathrm{and}\;H_{SRF,13}^{S}\left(\omega \right)$: For N=11 and zero-stared frequency, and by following the same procedures used for the derivation of $H_{SRF,11}^{C}\left(\omega \right)$, $H_{SRF,11}^{S}\left(\omega \right)$ can be written as:

(A13) $$H_{SRF,11}^{S}\left(\omega \right)=\frac{11+\sum_{\lambda =1}^{2}Bs_{\lambda }^{11}\left(\omega \right)}{11+\sum_{\lambda =1}^{10}Bs_{\lambda }^{11}\left(\omega \right)}$$

where

$$\begin{array}{cc} \; & Bs_{1}^{11}=4\left[\cos(k\pi )+\cos(3k\pi )+\cos(5k\pi )+\cos(7k\pi )+\cos(9k\pi )\right]\;\;\;\;\;\;\;\;\;\;\;\;\;\;\;\;\;\;\;\;\;\;\;\;\;\\ \; & \;\;\;\;\;\;\;\;\;\;\;\;\;\;\;\;\;\;\;\;\;\;\;\;\;\;\;\;\;\;\;\;\;\;\;\;\;\;\;\;\;\;\;\;\;\;\;\;\;\;\;\;\;\;\;\;\;\;\;\;\;\;\;\;\;\;\;\;\;\;\;\;\;\;\;\;\;\;\cos\left((10k\pi -\omega +\omega_{o})\right)\;,\\ \; & Bs_{2}^{11}=\left[2+4\cos(4k\pi )+4\cos(8k\pi )+4\cos(12k\pi )+4\cos(16k\pi )\right]\\ \; & \;\;\;\;\;\;\;\;\;\;\;\;\;\;\;\;\;\;\;\;\;\;\;\;\;\;\;\;\;\;\;\;\;\;\;\;\;\;\;\;\;\;\;\;\;\;\;\;\;\;\;\;\;\;\;\;\;\;\;\;\;\;\;\;\;\;\;\;\;\;\;\;\;\;\;\;\;\;\cos\left(2(10k\pi -\omega +\omega_{o})\right)\;,\\ \; & Bs_{3}^{11}=4\left[\cos(3k\pi )+\cos(9k\pi )+\cos(15k\pi )+\cos(21k\pi )\right]\cos\left(3(10k\pi -\omega +\omega_{o})\right)\;,\\ \; & Bs_{4}^{11}=\left[2+4\cos(8k\pi )+4\cos(16k\pi )+4\cos(24k\pi )\right]\cos\left(4(10k\pi -\omega +\omega_{o})\right)\;,\\ \; & Bs_{5}^{11}=4\left[\cos(5k\pi )+\cos(15k\pi )+\cos(25k\pi )\right]\cos\left(5(10k\pi -\omega +\omega_{o})\right)\;,\\ \; & Bs_{6}^{11}=\left[2+4\cos(14k\pi )+4\cos(24k\pi )\right]\cos\left(6(10k\pi -\omega +\omega_{o})\right)\;,\\ \; & Bs_{7}^{11}=4\left[\cos(7k\pi )+\cos(21k\pi )\right]\cos\left(7(10k\pi -\omega +\omega_{o})\right)\;,\\ \; & Bs_{8}^{11}=\left[2+4\cos(16k\pi )\right]\cos\left(8(10k\pi -\omega +\omega_{o})\right)\;,\\ \; & Bs_{9}^{11}=4\left[\cos(9k\pi )\right]\cos\left(9(10k\pi -\omega +\omega_{o})\right)\;,\;\mathrm{and}\\ \; & Bs_{10}^{11}=2\cos\left(10(10k\pi -\omega +\omega_{o})\right)\;. \end{array}$$

For N=13 and zero-stared frequency, and by following the same procedures used for derivation of $H_{SRF,11}^{C}\left(\omega \right)$, $H_{SRF,13}^{S}\left(\omega \right)$ can be written as:

(A14) $$H_{SRF,12}^{S}\left(\omega \right)=\frac{13+\sum_{\lambda =1}^{2}Bs_{\lambda }^{13}\left(\omega \right)}{13+\sum_{\lambda =1}^{12}Bs_{\lambda }^{13}\left(\omega \right)}$$

where

$$\begin{array}{cc} \; & Bs_{1}^{13}=4\left[\cos(k\pi )+\cos(3k\pi )+\cos(5k\pi )+\cos(7k\pi )+\cos(9k\pi )+\cos(11k\pi )\right]\;\;\;\;\;\;\;\;\;\;\;\;\;\;\;\;\;\;\;\;\;\;\;\;\;\;\;\;\;\;\;\\ \; & \;\;\;\;\;\;\;\;\;\;\;\;\;\;\;\;\;\;\;\;\;\;\;\;\;\;\;\;\;\;\;\;\;\;\;\;\;\;\;\;\;\;\;\;\;\;\;\;\;\;\;\;\;\;\;\;\;\;\;\;\;\;\;\;\;\;\;\;\;\;\;\;\;\;\;\;\cos\left((12k\pi -\omega +\omega_{o})\right)\;,\\ \; & Bs_{2}^{13}=\left[2+4\cos(4k\pi )+4\cos(8k\pi )+4\cos(12k\pi )+4\cos(16k\pi )+4\cos(20k\pi )\right]\\ \; & \;\;\;\;\;\;\;\;\;\;\;\;\;\;\;\;\;\;\;\;\;\;\;\;\;\;\;\;\;\;\;\;\;\;\;\;\;\;\;\;\;\;\;\;\;\;\;\;\;\;\;\;\;\;\;\;\;\;\;\;\;\;\;\;\;\;\;\;\;\;\;\;\;\;\;\;\cos\left(2(12k\pi -\omega +\omega_{o})\right)\;,\\ \; & Bs_{3}^{13}=4\left[\cos(3k\pi )+\cos(9k\pi )+\cos(15k\pi )+\cos(21k\pi )+\cos(27k\pi )\right]\\ \; & \;\;\;\;\;\;\;\;\;\;\;\;\;\;\;\;\;\;\;\;\;\;\;\;\;\;\;\;\;\;\;\;\;\;\;\;\;\;\;\;\;\;\;\;\;\;\;\;\;\;\;\;\;\;\;\;\;\;\;\;\;\;\;\;\;\;\;\;\;\;\;\;\;\;\;\;\cos\left(3(12k\pi -\omega +\omega_{o})\right)\;,\\ \; & Bs_{4}^{13}=\left[2+4\cos(8k\pi )+4\cos(16k\pi )+4\cos(24k\pi )+4\cos(32k\pi )\right]\\ \; & \;\;\;\;\;\;\;\;\;\;\;\;\;\;\;\;\;\;\;\;\;\;\;\;\;\;\;\;\;\;\;\;\;\;\;\;\;\;\;\;\;\;\;\;\;\;\;\;\;\;\;\;\;\;\;\;\;\;\;\;\;\;\;\;\;\;\;\;\;\;\;\;\;\;\;\;\cos\left(4(12k\pi -\omega +\omega_{o})\right)\;,\\ \; & Bs_{5}^{13}=4\left[\cos(5k\pi )+\cos(15k\pi )+\cos(25k\pi )+\cos(35k\pi )\right]\cos\left(5(12k\pi -\omega +\omega_{o})\right)\;,\\ \; & Bs_{6}^{13}=\left[2+4\cos(12k\pi )+4\cos(24k\pi )+4\cos(36k\pi )\right]\cos\left(6(12k\pi -\omega +\omega_{o})\right)\;,\\ \; & Bs_{7}^{13}=4\left[\cos(7k\pi )+\cos(21k\pi )+\cos(35k\pi )\right]\cos\left(7(12k\pi -\omega +\omega_{o})\right)\;,\\ \; & Bs_{8}^{13}=\left[2+4\cos(16k\pi )+4\cos(32k\pi )\right]\cos\left(8(12k\pi -\omega +\omega_{o})\right)\;,\\ \; & Bs_{9}^{13}=4\left[\cos(9k\pi )+\cos(27k\pi )\right]\cos\left(9(12k\pi -\omega +\omega_{o})\right)\;,\\ \; & Bs_{10}^{13}=\left[2+4\cos(20k\pi )\right]\cos\left(10(12k\pi -\omega +\omega_{o})\right)\;,\\ \; & Bs_{11}^{13}=4\left[\cos(11k\pi )\right]\cos\left(11(12k\pi -\omega +\omega_{o})\right)\;,\;\mathrm{and}\\ \; & Bs_{12}^{13}=2\cos\left(12(12k\pi -\omega +\omega_{o})\right)\;. \end{array}$$

**The general formula of**$H_{SRF,O}^{S}\left(\omega \right)$: A general formula of $H_{SRF,O}^{S}\left(\omega \right)$ is obtained from $H_{SRF,11}^{S}\left(\omega \right)$ and $H_{SRF,13}^{S}\left(\omega \right)$, given by Equations (A13) and (A14), respectively. After manipulations, $H_{SRF,O}^{S}\left(\omega \right)$ can be expressed as:

(A15) $$H_{SRF,O}^{S}\left(\omega \right)=\frac{H_{no}^{S}\left(\omega \right)}{H_{do}^{S}\left(\omega \right)}$$

where

$$\begin{array}{cc} \; & H_{no}^{S}\left(\omega \right)=N+\sum_{a=1}^{\frac{N_{u}}{2}+1}{FO}_{N}^{S}+\sum_{a=1}^{\frac{N_{u}}{2}}{FE}_{N}^{S}\;\;,\\ \; & H_{do}^{S}\left(\omega \right)=N+2\cos\left(\;\left(N-1\right)\;\;\Omega_{SN}\;\right)+\sum_{a=1}^{\frac{N-1}{2}}{FO}_{N}^{S}+\sum_{a=1}^{\frac{N-1}{2}-1}{FE}_{N}^{S}\;\;,\\ \; & {FO}_{N}^{S}=4\left(\sum_{b=1}^{\frac{N+1}{2}\;-\;\;a}O_{N}\right)\cos\left(\left(2a-1\right)\;\;\Omega_{SN}\right)\;,\;\;{FE}_{N}^{S}=2\left(\sum_{b=1}^{\frac{N-1}{2}\;-\;a}E_{N}+1\right)\cos\left(2a\;\;\Omega_{SN}\right)\;,\\ \; & \mathrm{and}\;\;\;\;\;\Omega_{SN}=\left(N-1\right)\pi \;k-\omega +\omega_{o}\;\;\;\;\;\;\;\;\;\;\;\;\;\;\;\;\;\;\;\;\;\;\;\;\;\;\;\;\;\;\;\;\;\;\;\;\;\;\;\;\;\;\;\;\;\;\;\;\;\;\;\;\;\;\;\;\;\;\;\;\;\;\;\;\;\;\;\;\;\;\;\;\;\;\;\;\;\;\;\;\;\;\;\;\;\;\;\;\;\;\;\;\;\;\;\;\;\;\;\;\;\;\;\;\;\;\;\;\;\;\;\;\;\;\;\;\;\;\;\;\;\;\;\;\;\;\;\;\;\;\;\;\;\;\;\;\;\;\;\;\; \end{array}$$

The general formula of $H_{SRF,O}^{C}\left(\omega \right)$ for odd *N*:

This combines $H_{SRF,O}^{C}\left(\omega \right)$ and $H_{SRF,O}^{S}\left(\omega \right)$, given by Equations (A12) and (A15), respectively. After manipulations, $H_{SRF,O}\left(\omega \right)$ can be expressed as:

(A16) $$H_{SRF,O}\left(\omega \right)=\frac{H_{no}\left(\omega \right)}{H_{do}\left(\omega \right)}$$

where

$$\begin{array}{cc} \; & H_{no}\left(\omega \right)=N+\sum_{a=1}^{\frac{N_{u}}{2}+1}{FO}_{N}+\sum_{a=1}^{\frac{N_{u}}{2}}{FE}_{N}\;\;,\;\;\;\;\;\;\;\;\;\;\;\;\;\;\;\;\;\;\;\;\;\;\;\;\;\;\;\;\;\;\;\;\;\;\;\;\;\;\;\;\;\;\;\;\;\;\;\;\\ \; & H_{do}\left(\omega \right)=N+2\cos\left(\left(N-1\right)\;\Omega_{o}\;\right)+\sum_{a=1}^{\frac{N-1}{2}}{FO}_{N}+\sum_{a=1}^{\frac{N-1}{2}-1}{FE}_{N}\;\;,\\ \; & {FO}_{N}=4\left(\sum_{b=1}^{\frac{N+1}{2}-a}O_{N}\right)\cos\left(\left(2a-1\right)\Omega_{o}\right)\;,\;\;{FE}_{N}=2\left(\sum_{b=1}^{\frac{N-1}{2}-a}E_{N}+1\right)\cos\left(2a\;\Omega_{o}\right)\;,\;\;\mathrm{and}\\ \; & \Omega_{o}=\left\{\begin{array}{c} \;\;\;\;\;\;\;\;\;\;\omega -\omega_{o}\;\;\;\;\;\;\;\;\;\;\;\;\;\;\;\;\;\;\;\;\;\;\;\;\;\;\;\mathrm{for}\;a\;\mathrm{zero-centered}\;\mathrm{frequency}\;\mathrm{LFM}\;\mathrm{waveform}.\\ \left(N-1\right)\pi k-\omega +\omega_{o}\;\;\;\;\mathrm{for}\;a\;\mathrm{zero-started}\;\mathrm{frequency}\;\mathrm{LFM}\;\mathrm{waveform}. \end{array}\right.. \end{array}$$

#### Appendix A.1.2. Derivation of $H_{SRF,E}\left(\omega \right)$ for even N:

$H_{SRF,E}\left(\omega \right)$ is obtained by considering the frequency responses of the SRF for an LFM waveform with zero-centered frequency and zero-started frequency, $H_{SRF,E}^{C}\left(\omega \right)$ and $H_{SRF,E}^{S}\left(\omega \right)$, respectively.

For even *N* and zero-centered frequency; $H_{SRF,E}^{C}\left(\omega \right)$:

$H_{SRF,10}^{C}\left(\omega \right)$, for N = 10, and $H_{SRF,12}^{C}\left(\omega \right)$, for N = 12, are used to get a general formula for $H_{SRF,E}^{C}\left(\omega \right)$.

$H_{SRF,10}^{C}\left(\omega \right)\;\mathrm{and}\;H_{SRF,12}^{C}\left(\omega \right)$: For N=10 and zero-centered frequency, and by following the same procedures used for derivation of $H_{SRF,11}^{C}\left(\omega \right)$, $H_{SRF,10}^{C}\left(\omega \right)$ can be written as:

(A17) $$H_{SRF,10}^{C}\left(\omega \right)=\frac{10+\sum_{\lambda =1}^{2}Bc_{\lambda }^{10}\left(\omega \right)}{10+\sum_{\lambda =1}^{9}Bc_{\lambda }^{10}\left(\omega \right)}$$

where

$$\begin{array}{cc} \; & Bc_{1}^{10}=\left[2+4\cos(2k\pi )+4\cos(4k\pi )+4\cos(6k\pi )+4\cos(8k\pi )\right]\cos\left((k\pi +\omega -\omega_{o})\right)\;,\\ \; & Bc_{2}^{10}=4\left[\cos(2k\pi )+\cos(6k\pi )+\cos(10k\pi )+\cos(14k\pi )\right]\cos\left(2(k\pi +\omega -\omega_{o})\right)\;,\\ \; & Bc_{3}^{10}=\left[2+4\cos(6k\pi )+4\cos(12k\pi )+4\cos(18k\pi )\right]\cos\left(3(k\pi +\omega -\omega_{o})\right)\;,\\ \; & Bc_{4}^{10}=4\left[\cos(4k\pi )+\cos(12k\pi )+\cos(20k\pi )\right]\cos\left(4(k\pi +\omega -\omega_{o})\right)\;,\\ \; & Bc_{5}^{10}=\left[2+4\cos(10k\pi )+4\cos(20k\pi )\right]\cos\left(5(k\pi +\omega -\omega_{o})\right)\;,\\ \; & Bc_{6}^{10}=4\left[\cos(6k\pi )+\cos(18k\pi )\right]\cos\left(6(k\pi +\omega -\omega_{o})\right)\;,\\ \; & Bc_{7}^{10}=\left[2+4\cos(14k\pi )\right]\cos\left(7(k\pi +\omega -\omega_{o})\right)\;,\\ \; & Bc_{8}^{10}=4\left[\cos(8k\pi )\right]\cos\left(8(k\pi +\omega -\omega_{o})\right)\;,\;\mathrm{and}\\ \; & Bc_{9}^{10}=2\cos\left(9(k\pi +\omega -\omega_{o})\right)\;. \end{array}$$

For N=12 and zero-centered frequency, and by following the same procedures used for derivation of $H_{SRF,11}^{C}\left(\omega \right)$, $H_{SRF,12}^{C}\left(\omega \right)$ can be written as:

(A18) $$H_{SRF,12}^{C}\left(\omega \right)=\frac{12+\sum_{\lambda =1}^{2}Bc_{\lambda }^{12}\left(\omega \right)}{12+\sum_{\lambda =1}^{11}Bc_{\lambda }^{12}\left(\omega \right)}$$

where

$$\begin{array}{cc} \; & Bc_{1}^{12}=\left[2+4\cos(2k\pi )+4\cos(4k\pi )+4\cos(6k\pi )+4\cos(8k\pi )+4\cos(10k\pi )\right]\;\;\;\;\;\;\;\;\;\;\;\;\;\;\;\;\;\;\;\;\;\;\;\;\;\;\;\;\\ \; & \;\;\;\;\;\;\;\;\;\;\;\;\;\;\;\;\;\;\;\;\;\;\;\;\;\;\;\;\;\;\;\;\;\;\;\;\;\;\;\;\;\;\;\;\;\;\;\;\;\;\;\;\;\;\;\;\;\;\;\;\;\;\;\;\;\;\;\;\;\;\;\;\;\;\;\;\;\;\;\;\cos\left((k\pi +\omega -\omega_{o})\right)\;,\\ \; & Bc_{2}^{12}=4\left[\cos(2k\pi )+\cos(6k\pi )+\cos(10k\pi )+\cos(14k\pi )+\cos(18k\pi )\right]\\ \; & \;\;\;\;\;\;\;\;\;\;\;\;\;\;\;\;\;\;\;\;\;\;\;\;\;\;\;\;\;\;\;\;\;\;\;\;\;\;\;\;\;\;\;\;\;\;\;\;\;\;\;\;\;\;\;\;\;\;\;\;\;\;\;\;\;\;\;\;\;\;\;\;\;\;\;\;\;\;\;\;\cos\left(2(k\pi +\omega -\omega_{o})\right)\;,\\ \; & Bc_{3}^{12}=\left[2+4\cos(6k\pi )+4\cos(12k\pi )+4\cos(18k\pi )+4\cos(24k\pi )\right]\\ \; & \;\;\;\;\;\;\;\;\;\;\;\;\;\;\;\;\;\;\;\;\;\;\;\;\;\;\;\;\;\;\;\;\;\;\;\;\;\;\;\;\;\;\;\;\;\;\;\;\;\;\;\;\;\;\;\;\;\;\;\;\;\;\;\;\;\;\;\;\;\;\;\;\;\;\;\;\;\;\;\;\cos\left(3(k\pi +\omega -\omega_{o})\right)\;,\\ \; & Bc_{4}^{12}=4\left[\cos(4k\pi )+\cos(12k\pi )+\cos(20k\pi )+\cos(28k\pi )\right]\cos\left(4(k\pi +\omega -\omega_{o})\right)\;,\\ \; & Bc_{5}^{12}=\left[2+4\cos(10k\pi )+4\cos(20k\pi )+4\cos(30k\pi )\right]\cos\left(5(k\pi +\omega -\omega_{o})\right)\;,\\ \; & Bc_{6}^{12}=4\left[\cos(6k\pi )+\cos(18k\pi )+\cos(30k\pi )\right]\cos\left(6(k\pi +\omega -\omega_{o})\right)\;,\\ \; & Bc_{7}^{12}=\left[2+4\cos(14k\pi )+4\cos(28k\pi )\right]\cos\left(7(k\pi +\omega -\omega_{o})\right)\;,\\ \; & Bc_{8}^{12}=4\left[\cos(8k\pi )+\cos(24k\pi )\right]\cos\left(8(k\pi +\omega -\omega_{o})\right)\;,\\ \; & Bc_{9}^{12}=\left[2+4\cos(18k\pi )\right]\cos\left(9(k\pi +\omega -\omega_{o})\right)\;,\\ \; & Bc_{10}^{12}=4\cos\left(10k\pi \right)\cos\left(10(k\pi +\omega -\omega_{o})\right)\;,\;\mathrm{and}\\ \; & Bc_{11}^{12}=2\cos\left(11(k\pi +\omega -\omega_{o})\right)\;. \end{array}$$

**The general formula of**$H_{SRF,E}^{C}\left(\omega \right)$: A general formula of $H_{SRF,E}^{C}\left(\omega \right)$ is obtained from combining $H_{SRF,10}^{C}\left(\omega \right)$ and $H_{SRF,12}^{C}\left(\omega \right)$, given by Equations (A17) and (A18), respectively. After manipulations, $H_{SRF,E}^{C}\left(\omega \right)$ can be expressed as:

(A19) $$H_{SRF,E}^{C}\left(\omega \right)=\frac{H_{ne}^{C}\left(\omega \right)}{H_{de}^{C}\left(\omega \right)}$$

where

$$\begin{array}{cc} \; & H_{ne}^{C}\left(\omega \right)=N+\sum_{a=1}^{\frac{N_{u}}{2}}{FG}_{N}^{C}+\sum_{a=1}^{\frac{N_{u}}{2}}{FP}_{N}^{C}\;\;,\\ \; & H_{de}^{C}\left(\omega \right)=N+2\cos\left(\left(N-1\right)\Omega_{CNe}\right)+\sum_{a=1}^{\frac{N}{2}-1}{FG}_{N}^{C}+\sum_{a=1}^{\frac{N}{2}-1}{FP}_{N}^{C}\;\;,\\ \; & {FG}_{N}^{C}=\left(4\sum_{b=1}^{\frac{N}{2}-a}G_{N}+2\right)\cos\left[\left(2a-1\right)\Omega_{CNe}\right]\;,\;\;{FP}_{N}^{C}=4\left(\sum_{b=1}^{\frac{N}{2}-a}P_{N}\right)\cos\left(2a\Omega_{CNe}\right)\;,\\ \; & \Omega_{CNe}=k\pi +\omega -\omega_{o},\;\;\;G_{N}=\cos\left(\left(2a-1\right)\left(2\pi bk\right)\right),\;\;\;\mathrm{and}\;\;\;P_{N}=\cos\left(2a\left(2b-1\right)\pi k\right).\;\;\;\;\;\;\;\;\;\;\;\;\;\;\;\;\;\;\;\;\;\;\;\;\;\; \end{array}$$

For even *N* and zero-started frequency; $H_{SRF,10}^{S}\left(\omega \right)$:

Consider two values of *N*; 10 and 12, to get $H_{SRF,10}^{S}\left(\omega \right)$ and $H_{SRF,12}^{S}\left(\omega \right)$, respectively, which can be used to deduce a general form of $H_{SRF,E}^{S}\left(\omega \right)$.

$H_{SRF,10}^{S}\left(\omega \right)\;\mathrm{and}\;H_{SRF,12}^{S}\left(\omega \right)$: For N=10 and zero-stared frequency, and by following the same procedures used for derivation of $H_{SRF,11}^{C}\left(\omega \right)$, $H_{SRF,10}^{S}\left(\omega \right)$ can be written as:

(A20) $$H_{SRF,10}^{S}\left(\omega \right)=\frac{10+\sum_{\lambda =1}^{2}Bs_{\lambda }^{10}\left(\omega \right)}{10+\sum_{\lambda =1}^{9}Bs_{\lambda }^{10}\left(\omega \right)}$$

where

$$\begin{array}{cc} \; & Bs_{1}^{10}=\left[2+4\cos(2k\pi )+4\cos(4k\pi )+4\cos(6k\pi )+4\cos(8k\pi )\right]\cos\left((9k\pi +\omega -\omega_{o})\right)\;,\\ \; & Bs_{2}^{10}=4\left[\cos(2k\pi )+\cos(6k\pi )+\cos(10k\pi )+\cos(14k\pi )\right]\cos\left(2(9k\pi +\omega -\omega_{o})\right)\;,\\ \; & Bs_{3}^{10}=\left[2+4\cos(6k\pi )+4\cos(12k\pi )+4\cos(18k\pi )\right]\cos\left(3(9k\pi +\omega -\omega_{o})\right)\;,\\ \; & Bs_{4}^{10}=4\left[\cos(4k\pi )+\cos(12k\pi )+\cos(20k\pi )\right]\cos\left(4(9k\pi +\omega -\omega_{o})\right)\;,\\ \; & Bs_{5}^{10}=\left[2+4\cos(10k\pi )+4\cos(20k\pi )\right]\cos\left(5(9k\pi +\omega -\omega_{o})\right)\;,\\ \; & Bs_{6}^{10}=4\left[\cos(6k\pi )+\cos(18k\pi )\right]\cos\left(6(9k\pi +\omega -\omega_{o})\right)\;,\\ \; & Bs_{7}^{10}=\left[2+4\cos(14k\pi )\right]\cos\left(7(9k\pi +\omega -\omega_{o})\right)\;,\\ \; & Bs_{8}^{10}=4\left[\cos(8k\pi )\right]\cos\left(8(9k\pi +\omega -\omega_{o})\right)\;,\;\mathrm{and}\\ \; & Bs_{9}^{10}=2\cos\left(9(9k\pi +\omega -\omega_{o})\right)\;. \end{array}$$

For N=12 and zero-stared frequency, and by following the same procedures used for derivation of $H_{SRF,11}^{C}\left(\omega \right)$, $H_{SRF,12}^{S}\left(\omega \right)$ can be written as:

(A21) $$H_{SRF,12}^{S}\left(\omega \right)=\frac{12+\sum_{\lambda =1}^{2}Bs_{\lambda }^{12}\left(\omega \right)}{12+\sum_{\lambda =1}^{11}Bs_{\lambda }^{12}\left(\omega \right)}$$

where

$$\begin{array}{cc} \; & Bs_{1}^{12}=\left[2+4\cos(2k\pi )+4\cos(4k\pi )+4\cos(6k\pi )+4\cos(8k\pi )+4\cos(10k\pi )\right]\;\;\;\;\;\;\;\;\;\;\;\;\;\;\;\;\;\;\;\;\;\;\;\;\;\;\;\;\;\;\;\;\;\;\;\;\;\;\;\;\;\;\;\;\;\;\;\;\;\;\\ \; & \;\;\;\;\;\;\;\;\;\;\;\;\;\;\;\;\;\;\;\;\;\;\;\;\;\;\;\;\;\;\;\;\;\;\;\;\;\;\;\;\;\;\;\;\;\;\;\;\;\;\;\;\;\;\;\;\;\;\;\;\;\;\;\;\;\;\;\;\;\;\;\;\cos\left((11k\pi -\omega +\omega_{o})\right)\;,\\ \; & Bs_{2}^{12}=4\left[\cos(2k\pi )+\cos(6k\pi )+\cos(10k\pi )+\cos(14k\pi )+\cos(18k\pi )\right]\\ \; & \;\;\;\;\;\;\;\;\;\;\;\;\;\;\;\;\;\;\;\;\;\;\;\;\;\;\;\;\;\;\;\;\;\;\;\;\;\;\;\;\;\;\;\;\;\;\;\;\;\;\;\;\;\;\;\;\;\;\;\;\;\;\;\;\;\;\;\;\;\;\;\;\cos\left(2(11k\pi -\omega +\omega_{o})\right)\;,\\ \; & Bs_{3}^{12}=\left[2+4\cos(6k\pi )+4\cos(12k\pi )+4\cos(18k\pi )+4\cos(24k\pi )\right]\\ \; & \;\;\;\;\;\;\;\;\;\;\;\;\;\;\;\;\;\;\;\;\;\;\;\;\;\;\;\;\;\;\;\;\;\;\;\;\;\;\;\;\;\;\;\;\;\;\;\;\;\;\;\;\;\;\;\;\;\;\;\;\;\;\;\;\;\;\;\;\;\;\;\;\cos\left(3(11k\pi -\omega +\omega_{o})\right)\;,\\ \; & Bs_{4}^{12}=4\left[\cos(4k\pi )+\cos(12k\pi )+\cos(20k\pi )+\cos(28k\pi )\right]\cos\left(4(11k\pi -\omega +\omega_{o})\right)\;,\\ \; & Bs_{5}^{12}=\left[2+4\cos(10k\pi )+4\cos(20k\pi )+4\cos(30k\pi )\right]\cos\left(5(11k\pi -\omega +\omega_{o})\right)\;,\\ \; & Bs_{6}^{12}=4\left[\cos(6k\pi )+\cos(18k\pi )+\cos(30k\pi )\right]\cos\left(6(11k\pi -\omega +\omega_{o})\right)\;,\\ \; & Bs_{7}^{12}=\left[2+4\cos(14k\pi )+4\cos(28k\pi )\right]\cos\left(7(11k\pi -\omega +\omega_{o})\right)\;,\\ \; & Bs_{8}^{12}=4\left[\cos(8k\pi )+\cos(24k\pi )\right]\cos\left(8(11k\pi -\omega +\omega_{o})\right)\;,\\ \; & Bs_{9}^{12}=\left[2+4\cos(18k\pi )\right]\cos\left(9(11k\pi -\omega +\omega_{o})\right)\;,\\ \; & Bs_{10}^{12}=4\cos\left(10k\pi \right)\cos\left(10(11k\pi -\omega +\omega_{o})\right)\;,\;\mathrm{and}\\ \; & Bs_{11}^{12}=2\cos\left(11(11k\pi -\omega +\omega_{o})\right)\;. \end{array}$$

**The general formula of**$H_{SRF,E}^{S}\left(\omega \right)$: A general formula of $H_{SRF,E}^{S}\left(\omega \right)$ can be obtained from combining $H_{SRF,10}^{S}\left(\omega \right)$ and $H_{SRF,12}^{S}\left(\omega \right)$, given by Equations (A20) and (A21), respectively. After manipulations, $H_{SRF,E}^{S}\left(\omega \right)$ can be expressed as:

(A22) $$H_{SRF,E}^{S}\left(\omega \right)=\frac{H_{ne}^{S}\left(\omega \right)}{H_{de}^{S}\left(\omega \right)}$$

where

$$\begin{array}{cc} \; & H_{ne}^{S}\left(\omega \right)=N+\sum_{a=1}^{\frac{N_{u}}{2}}{FG}_{N}^{S}+\sum_{a=1}^{\frac{N_{u}}{2}}{FP}_{N}^{S}\;\;,\\ \; & H_{de}^{S}\left(\omega \right)=N+2\cos\left(\left(N-1\right)\;\Omega_{SN}\right)+\sum_{a=1}^{\frac{N}{2}-1}{FG}_{N}^{S}+\sum_{a=1}^{\frac{N}{2}-1}{FP}_{N}^{S}\;\;,\\ \; & {FG}_{N}^{S}=\left(4\sum_{b=1}^{\frac{N}{2}-a}G_{N}+2\right)\cos\left(\left(2a-1\right)\Omega_{SN}\right)\;,\;and\;\;{FP}_{N}^{S}=4\left(\sum_{b=1}^{\frac{N}{2}-a}P_{N}\right)\cos\left(2a\Omega_{SN}\right)\;. \end{array}$$

The general formula of $H_{SRF,E}\left(\omega \right)$ for even *N*:

This combines $H_{SRF,E}^{C}\left(\omega \right)$ and $H_{SRF,E}^{S}\left(\omega \right)$, given by Equations (A19) and (A22), respectively. After manipulations, $H_{SRF,E}\left(\omega \right)$ can be expressed as:

(A23) $$H_{SRF,E}\left(\omega \right)=\frac{H_{ne}\left(\omega \right)}{H_{de}\left(\omega \right)}\;\;\;\;\;\;$$

where

$$\begin{array}{cc} \; & H_{ne}\left(\omega \right)=N+\sum_{a=1}^{\frac{N_{u}}{2}}{FG}_{N}+\sum_{a=1}^{\frac{N_{u}}{2}}{FP}_{N}\;\;,\;\;\;\;\;\;\;\;\;\;\;\;\;\;\;\;\;\;\;\;\;\;\;\;\;\;\;\;\;\;\;\;\;\;\;\;\;\;\;\;\;\;\;\;\;\;\;\;\;\;\;\;\;\;\;\;\;\;\;\;\;\;\;\;\;\;\;\;\;\;\;\;\;\;\;\;\;\;\;\;\;\;\;\;\;\;\;\;\;\;\;\;\;\;\;\;\;\;\;\;\;\\ \; & H_{de}\left(\omega \right)=N+2\cos\left(\left(N-1\right)\;\Omega_{e}\right)+\sum_{a=1}^{\frac{N}{2}-1}{FG}_{N}+\sum_{a=1}^{\frac{N}{2}-1}{FP}_{N}\;\;,\\ \; & {FG}_{N}=\left(4\sum_{b=1}^{\frac{N}{2}-a}G_{N}+2\right)\cos\left(\left(2a-1\right)\Omega_{e}\right)\;\;,\;{FP}_{N}=4\left(\sum_{b=1}^{\frac{N}{2}-a}P_{N}\right)\cos\left(2a\Omega_{e}\right)\;,\;\;\mathrm{and}\\ \; & \Omega_{e}=\left\{\begin{array}{c} \;\;\;\;k\pi +\omega -\omega_{o}\;\;\;\;\;\;\;\;\;\;\;\;\;\;\;\;\;\;\mathrm{for}\;\mathrm{zero-centered}\;\mathrm{frequency}\;\mathrm{LFM}\;\mathrm{waveform}\\ \left(N-1\right)\pi \;k-\omega +\omega_{o}\;\;\;\;\;\mathrm{for}\;\mathrm{zero-started}\;\mathrm{frequency}\;\mathrm{LFM}\;\mathrm{waveform} \end{array}\right. \end{array}$$
